# Multiphase Interfacial Regulation Based on Hierarchical Porous Molybdenum Selenide to Build Anticorrosive and Multiband Tailorable Absorbers

**DOI:** 10.1007/s40820-023-01212-4

**Published:** 2023-11-06

**Authors:** Tianbao Zhao, Zirui Jia, Jinkun Liu, Yan Zhang, Guanglei Wu, Pengfei Yin

**Affiliations:** 1 College of Science, Sichuan Agricultural University, Ya’an, 625014 People’s Republic of China; 2 Institute of Materials for Energy and Environment, State Key Laboratory of Bio-Fibers and Eco-Textiles, College of Materials Science and Engineering, Qingdao University, Qingdao, 266071 People’s Republic of China; 3 College of Chemistry and Chemical Engineering, Qingdao University, Qingdao, 266071 People’s Republic of China

**Keywords:** Interfacial engineering, Anticorrosion, Multiband, Electromagnetic wave absorber

## Abstract

Electromagnetic wave (EMW) absorbing materials have an irreplaceable position in the field of military stealth as well as in the field of electromagnetic pollution control. And in order to cope with the complex electromagnetic environment, the design of multifunctional and multiband high efficiency EMW absorbers remains a tremendous challenge. In this work, we designed a three-dimensional porous structure via the salt melt synthesis strategy to optimize the impedance matching of the absorber. Also, through interfacial engineering, a molybdenum carbide transition layer was introduced between the molybdenum selenide nanoparticles and the three-dimensional porous carbon matrix to improve the absorption behavior of the absorber. The analysis indicates that the number and components of the heterogeneous interfaces have a significant impact on the EMW absorption performance of the absorber due to mechanisms such as interfacial polarization and conduction loss introduced by interfacial engineering. Wherein, the prepared MoSe_2_/MoC/PNC composites showed excellent EMW absorption performance in *C*, *X*, and *K*_*u*_ bands, especially exhibiting a reflection loss of − 59.09 dB and an effective absorption bandwidth of 6.96 GHz at 1.9 mm. The coordination between structure and components endows the absorber with strong absorption, broad bandwidth, thin thickness, and multi-frequency absorption characteristics. Remarkably, it can effectively reinforce the marine anticorrosion property of the epoxy resin coating on Q235 steel substrate. This study contributes to a deeper understanding of the relationship between interfacial engineering and the performance of EMW absorbers, and provides a reference for the design of multifunctional, multiband EMW absorption materials.

**Supplementary Information:**

The online version contains supplementary material available at 10.1007/s40820-023-01212-4.

## Highlights

- The hierarchical porous structure is regulated by various species of PVP to achieve impedance matching.
- Interfacial engineering boosts conductivity and constructs a multiband (C, X, Ku) tunable electromagnetic wave absorber.
- Hierarchical porous molybdenum selenide/epoxy coating exhibits marine anticorrosion capability.

**Supplementary Information:**

The online version contains supplementary material available at 10.1007/s40820-023-01212-4.

## Graphical abstract

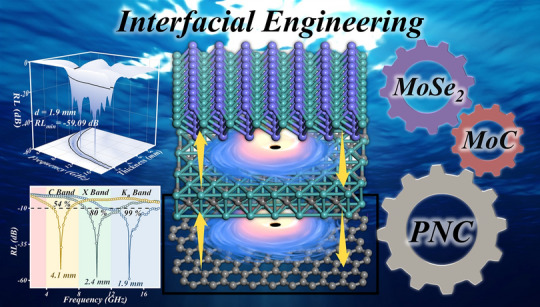

## Introduction

With the rapid development of wireless communication technology, especially the booming growth of 5G and human over-reliance on various intelligent devices, electromagnetic radiation is ubiquitous, and the electromagnetic pollution problem we are facing is becoming increasingly serious [1–3]. In one way, overabundance electromagnetic waves may provoke electromagnetic disturbance, impairing signal communication and even hindering the normal functioning of electronic and intelligent facilities [4–6]. More worryingly, researches indicate that exposure to high densities of electromagnetic waves negatively damages health and raises the threat of disease [7–9]. Developing electromagnetic wave (EMW) absorption materials is widely regarded as a direct and effective way to alleviate electromagnetic pollution [10–12]. EMW absorption materials are materials that are sensitive to EMWs and can absorb EMWs to a certain extent [13–15]. They can not only mitigate electromagnetic interference on equipment and the potential harm to human health but also play a crucial role in improving stealth technology in the military [16–18]. Regrettably, designing efficient, multifunctional, and multiband EMW absorbers to cope with complex electromagnetic environments remains a tremendous challenge [19–21].

Undoubtedly, microstructure design is a crucial factor in the development of high-performance electromagnetic absorbers [22, 23]. For example, reasonable microstructures such as cavities or shell layers can adjust the complex permittivity while achieving lightweight material [24–26]. Moreover, the existence of internal voids can introduce multiple reflections and scattering effects, which can improve the electromagnetic attenuation capability and impedance matching of the absorber [27–29]. Researchers have long been devoted to designing and fabricating special structure absorbers, such as hollow, core–shell, yolk-shell, multi-shell, and porous structures [30–32]. Among them, porous carbon materials based on porous structure have been widely used in the field of EMW absorption due to their excellent electrical conductivity, adjustable dielectric performance, low density, light mass, and other factors that have been more profoundly studied [33]. In general, porous carbon materials can be fabricated by chemical/physical activation methods and various template methods [34]. For instance, porous carbon can be derived from biomass materials, ZIF, MOF, etc., and prepared using templates such as polymers and silica. However, the disadvantages of these typical methods are also obvious, such as high cost, complicated process, time-consuming, harsh conditions, and difficult recovery. Fortunately, salt melt synthesis (SMS) can overcome the above disadvantages to a large extent [35]. First, salt templates are more readily available due to their large reserves and variety compared to typical template methods. Moreover, since most of the salts are water soluble, the products are easily separated. Furthermore, many salts are environmentally friendly, non-toxic, and can be recycled and reused [36]. Inspired by this, designing porous structures using the SMS strategy may be a more sensible choice.

Transition metal dichalcogenides (TMDCs) are two-dimensional materials with a layered structure that have the advantages of unique morphology, a narrow band gap, and outstanding dielectric properties and are popular materials for building EMW absorbers [37]. As typical TMDCs, MoSe_2_ exhibits more metallic character and stability compared to the extensively studied MoS_2_ and possesses a narrower bandgap (1.33–1.72 eV) and higher conductivity [38]. In addition, and more importantly, the strength of the Mo-Se bond is weaker due to the weaker binding of selenium atoms to electrons, which makes it easier for MoSe_2_ to form dissipative currents to attenuate EMWs. For instance, Ji et al. precisely tuned the morphological structure of flower-like MoSe_2_ to implement favorable absorption performance in multiple frequency bands [39].

Interfacial engineering can introduce various defects into materials, such as vacancies, heteroatom doping, dislocations, and twinning [40]. More critically, the interfacial polarization induced by the heterogeneous interface enhances the dielectric loss capability of the absorber, which further promotes the efficient absorption of EMWs. Jia et al. introduced multiple heterogeneous interfaces via subtly manipulating the MoO_2_/C sulfidation level, attesting to interfacial engineering as effective strategy to improve the EMW absorption capacity [41]. Previous experiments and theoretical calculations have evidenced that interfacial coupling between MoSe_2_ and carbon substrates can be performed through Mo–C or Mo–O–C chemical bonds, which effectively enhance electrical conductivity and structural stability [42, 43]. Therefore, interfacial engineering is a promising avenue for enhancing the EMW absorption capacity and stability of absorbers.

Herein, we have successfully synthesized a series of molybdenum-based hierarchical porous nitrogen-doped carbon (PNC) composites with different heterogeneous interfacial structures by freeze drying, high-temperature pyrolysis, and washing using the SMS strategy. The optimization of the performance of EMW absorbers by interfacial engineering is explored in depth through multifaceted characterization as well as testing of the three-electrode system and electromagnetic parameters. Typically, by introducing a highly conductive and stable MoC transition layer between MoSe_2_ particles and PNC, the prepared MoSe_2_/MoC/PNC composites have significantly improved EMW absorption performance and stability, resulting in superior reflection loss (RL) and effective absorption bandwidth (EAB) in *C*, *X*, and *K*_*u*_ bands. In particular, the composite obtained − 59.09 dB RL and 6.96 GHz EAB at a thickness of 1.9 mm, and more importantly, it also effectively reinforces the marine corrosion protection of the epoxy composite coating based on Q235 steel, thus effectively dealing with complex electromagnetic environments.

## Experimental

### Chemicals and Materials

Ammonium hepta-molybdate tetrahydrate ((NH_4_)_6_Mo_7_O_24_·4H_2_O), sodium chloride (NaCl), polyvinylpyrrolidone ((C_6_H_9_NO)_*n*_, PVP), selenium powder, epoxy resin, polyamide curing agent, xylene, n-butanol, and ethanol (C_2_H_5_OH) were purchased from Aladdin. All reagents are of analytical grade (AR) and can be used directly without further purification.

### Preparation of Precursors

First, 0.3 g NaCl, 0.4 g (NH_4_)_6_Mo_7_O_24_·4H_2_O and 1.0 g PVP-K*x* (*x* = 30, 60, 90) were added to 25 mL distilled water, which was stirred and sonicated to fully dissolve and obtain a clear solution. Next, the resulting solution was rapidly frozen with liquid nitrogen. Finally, it was vacuum freeze-dried for at least 48 h to obtain the precursors.

### Preparation of Hierarchical Porous Molybdenum Selenide Composites

Firstly, 0.3 g of precursor was thoroughly mixed with 0.06 g of selenium powder, and the mixture was transferred to a porcelain boat. Then, it was heat treated at 600 °C for 3 h under Ar atmosphere at a heating rate of 4 °C min^−1^ and a gas flow rate of 80 mL min^−1^ to obtain NaCl@MoSe_2_/MoO_2_/PNC-x composites. Next, the NaCl@MoSe_2_/MoO_2_/PNC-x composites were added to ultrapure water and stirred vigorously for 0.5 h. The resulting solutions were then washed by filtration with ethanol and ultrapure water for several times, and finally dried in a vacuum oven at 65 °C for 12 h to obtain MoSe_2_/MoO_2_/PNC-x composites with three-dimensional porous structure. By varying the heat treatment temperature to 700 and 800 °C, MoSe_2_/PNC-x and MoSe_2_/MoC/PNC-x can be obtained eventually. In addition, without adding selenium powder, MoO_2_/PNC-x and Mo_2_C/PNC-x can eventually be obtained at heat treatment temperatures of 600 and 800 °C, respectively.

### Preparation of Composite Coatings

Five g of epoxy resin was completely dissolved with 10 mL of ethanol, and then 50 mg of sample was added as filler. The mixture was homogeneously dispersed by ultrasonic stirring. The ethanol is then removed by stirring at 50 °C. A solvent mixture containing xylene and n-butanol is added to the epoxy resin containing the filler and stirred until well mixed. Then, polyamide curing agent is added and stirred for 30 min. To remove the air bubbles generated during the coating preparation, the well-mixed solution was transferred to a vacuum drying oven and placed. Finally, the obtained mixture was evenly coated on the clean Q235 steel surface and left at room temperature for 3 days. Ultimately, the corresponding composite coating can be obtained depending on the filler. In addition, a pure epoxy resin coating was prepared as a comparison.

### Characterization

Powder X-ray diffraction (XRD, Rigaku Ultima IV, Cu-Ka radiation (*λ* = 0.15418)). Raman spectra of the samples were acquired using a Renishaw InVia Plus micro-Raman spectroscopy system equipped with a 50 mW 532 mm DPSS laser. The morphology and elemental mapping of the samples were observed with a field emission scanning electron microscope (SEM, JEOL JSM-7800F), and the lattice spacing of the samples was observed with a transmission electron microscope (TEM, JEOL JEM-2100). Thermogravimetric analysis (TGA) was performed on an SDT Q600 analyzer under an atmosphere of air with a ramp-up rate of 10 °C min^−1^ from room temperature to 800 °C. The porous structure was characterized by adsorption–desorption of N_2_ on a Quantachrome Autosorb iQ3. The specific surface area was calculated according to the Brunauer–Emmett–Teller (BET) method, and the pore size distribution was estimated by the density functional theory (DFT) method. The distribution of elements on the surface of the composites was characterized by X-ray photoelectron spectroscopy (XPS) on a Thermo Fisher ESCALAB 250Xi energy spectrometer using an Al Ka X-ray source (1486.6 eV).

### Electromagnetic Parameters and Anticorrosion Performance Test of Coatings

The prepared sample powder was uniformly mixed with paraffin wax (sample powder mass ratio of 27.5 wt%), and the mixed sample was pressed into a circular shape with a thickness of about 2 mm through a cylindrical mold with an outer diameter of 7 mm and an inner diameter of 3.04 mm. The electromagnetic parameters complex permittivity *ε*_*r*_ (*ε*_*r*_ = *ε′–jε″*) and complex permeability *μ*_*r*_ = (*μ*_*r*_ = *μ′–jμ″*) were measured by the coaxial line method on a vector network analyzer (VNA, Agilent N5222A) in the frequency range of 2–18 GHz. the RL values can be calculated according to transmission line theory by the following equation [44–46]:

1 $$Z_{{{\text{in}}}} = Z_{0} \sqrt {\frac{{\mu_{r} }}{{\varepsilon_{r} }}} \tanh \left( {j\frac{2\pi fd}{c}\sqrt {\varepsilon_{r} \mu_{r} } } \right)$$

2 $$RL\;({\text{dB}}) = 20\log \frac{{Z_{{{\text{in}}}} - Z_{0} }}{{Z_{{{\text{in}}}} + Z_{0} }}$$

where *Z*_in_ and *Z*_0_ denote the input impedance and free-space characteristic impedance of the standard absorbing material, respectively, *f* denotes the frequency of the EMW, *d* denotes the thickness of the sample, and *c* denotes the speed of the EMW in free-space [47, 48].

The electrochemical corrosion experiments of all samples were measured with an electrochemical workstation CHI 760E in seawater solution through a typical three-electrode system. Where a platinum sheet was used as counter electrode, Ag/AgCl as reference electrode and coated electrodes as working electrodes. (The seawater solution used was taken from the local coastal area of Qingdao). The open circuit potential (OCP) behavior was recorded and electrochemical impedance spectroscopy (EIS) measurements were performed in the frequency range of 10^–2^–10^5^ Hz.

## Results and Discussion

### Characterization

**Fig. 1:**
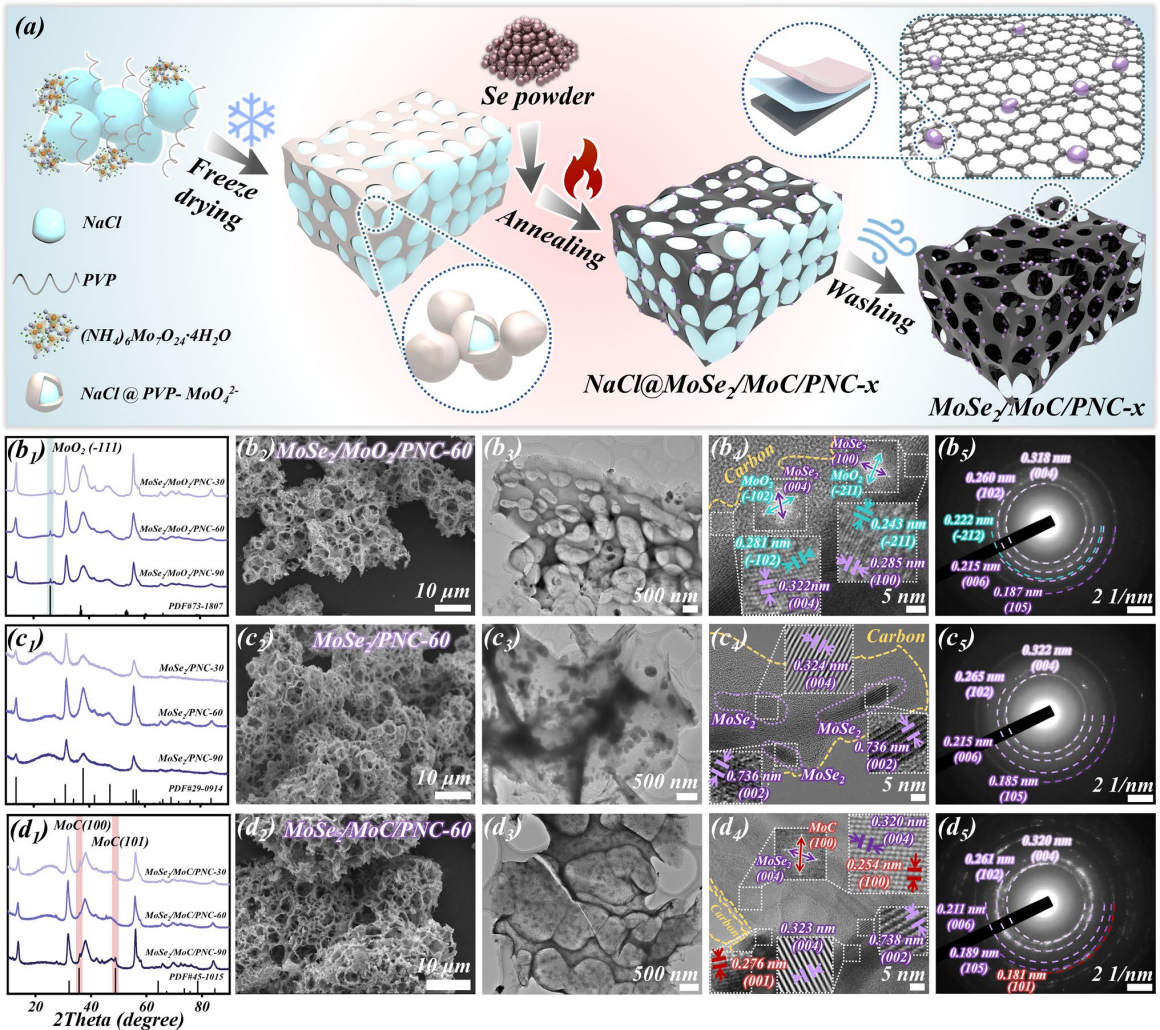
**a** Schematic diagram of preparation of hierarchical porous molybdenum selenide, **b**_**1**_**–d**_**1**_ XRD patterns of MoSe_2_/MoO_2_/PNC-x, MoSe_2_/PNC-x, and MoSe_2_/MoC/PNC-x, **b**_**2**_**–d**_**2**_ SEM images, **b**_**3**_**–d**_**3**_ TEM images, **b**_**4**_**–d**_**4**_ HRTEM images, **b**_**5**_**–d**_**5**_ SAED images of MoSe_2_/MoO_2_/PNC-60, MoSe_2_/PNC-60, and MoSe_2_/MoC/PNC-60

The preparation process of a series of PNC composites with different molybdenum-based doping is shown in Fig. 1a. The main experimental procedure is divided into three parts. First, NaCl was used as the salt template in the SMS strategy, ammonium molybdate tetrahydrate as the molybdenum source, and different PVP as the nitrogen-rich carbon skeleton source. Meanwhile, PVP was used as a nonionic surfactant to polymerize with the molybdate ion (MoO_4_^2−^) in solution. After freeze drying, the NaCl in solution recrystallizes and forms precursors with a three-dimensional structure under the encapsulation of PVP-MoO_4_^2−^. During the subsequent heat treatment, PVP is transformed into a nitrogen-doped carbon skeleton. And due to the presence of selenium powder, the molybdenum precursors were transformed into molybdenum selenide nanoparticles, which were grown on the carbon matrix. Finally, the NaCl salt template was removed by washing and its hierarchical porous structure was finally formed.

Typically, PVPs with different molecular weight sizes differ in solubility, viscosity, stability, hydrophilicity, and intermolecular forces [49]. Therefore, by using different species of PVP, the final hierarchical porous structure obtained also differs, which affects the impedance matching performance. As shown in Figs. S1–S3, the prepared MoSe_2_/MoC/PNC-x composites have typical hierarchical porous characteristics, and the uniform distribution of Mo, Se, C, N, and O elements across the PNC as observed in their EDS element mapping. The presence of N elements in them proves that N atoms are successfully doped into the carbon matrix. More importantly, it can be observed that the final PNC skeleton obtained using PVP-K30 as the carbon source is thicker and the porous structure is mostly formed only on the surface without penetrating deep into the carbon matrix. In contrast, the PNC skeleton finally obtained by using PVP-K60 as the carbon source was significantly optimized, and dense and deep pores could be observed to be uniformly distributed on the carbon matrix. In addition, the PNC skeleton formed using PVP-K90 as the carbon source was thinner and the distribution of holes was more intensive. This strongly demonstrates that the specie of PVP has a modifying effect on the porous structure of PNC.

Figure 1b1–d1 shows the XRD patterns of a series of selenized samples obtained using different species of PVP and under different heat treatment conditions. As shown in Fig. 1b1, several diffraction peaks belonging to MoSe_2_ (JCPDS No. 29-0914) can be observed near 17.8°, 31.8°, 37.9°, and 56.0°. In addition, a peak of lower intensity can be observed near its 26.1°, which corresponded to the (− 111) crystal plane of MoO_2_ (JCPDS No. 73-1807), indicating the presence of trace amounts of MoO_2_ in the sample. The results indicate that the sample is incompletely selenized at 600 °C and the material formed is MoSe_2_/MoO_2_/PNC-x. When the heat treatment temperature was 700 °C, the sample was completely selenized and it was observed that all the diffraction peaks corresponded perfectly to the standard PDF card of MoSe_2_, generating MoSe_2_/PNC-x, as shown in Fig. 1c1. And when the heat treatment temperature is 800 °C, two weak peaks near 36.0° and 48.9° can be observed in the XRD pattern of its product (Fig. 1d1), corresponded to the (100) and (101) crystal planes of the γ-phase molybdenum carbide (MoC, JCPDS No. 45-1015), indicating the generation of a small amount of MoC, implying that the MoSe_2_/MoC/PNC-x was successfully prepared. In Fig. S4, it is clearly observed that the intensity of the corresponding diffraction peak of MoSe_2_ decreased with increase in heat treatment temperature. In particular, the intensity of the peak located at 17.8° is significantly reduced, which indicates an increase in the number of defects along the [002] direction in its (002) crystal plane [42]. As a comparison, MoO_2_/PNC-x and Mo_2_C/PNC-x were also prepared in the absence of selenium powder (their corresponding XRD patterns are shown in Figs. S5 and S6). It is worth noting that the Mo_2_C generated at this time is of the β-phase (JCPDS No. 35-0787), which is a different phase from the MoC in MoSe_2_/MoC/PNC-x. The above results revealed that molybdenum-based nanoparticles with different components can be grown on PNC substrates by modulating the heat treatment conditions. With the increase in selenization, all MoO_2_ was gradually converted to MoSe_2_ and MoC was generated at high temperature. The obtained EDS data can also support this result (Fig. S7). As shown in Table S1, the mass ratio of each element was obtained, and the atomic ratio of Mo and Se elements (Mo/Se at.%) could be obtained by conversion. Among them, Mo/Se at.% in both MoSe_2_/MoO_2_/PNC-60 and MoSe_2_/MoC/PNC-60 are slightly higher than 50%, indicating the presence of a few other compounds of Mo in addition to MoSe_2_. And the Mo/Se at.% of MoSe_2_/PNC-60 was 50.23%, which again proved that the sample was completely selenized.

The morphology of MoSe_2_/MoO_2_/PNC-60, MoSe_2_/PNC-60 and MoSe_2_/MoC/PNC-60 is almost indistinguishable from the SEM images (Fig. 1b2–d2) and EDS elemental mapping (Figs. S2, S8, and S9) of the samples obtained under different annealing conditions, all showing a uniform dense porous structure and the presence of molybdenum selenide nanoparticles on the surface. Similar porous structures can also be observed in the SEM images of MoO_2_/PNC-60 and Mo_2_C/PNC-60 (Figs. S10 and S11), but the surfaces are smoother compared to the selenized samples. This revealed that the heat treatment temperature has little impact on the morphology of PNC, but the molybdenum selenide nanoparticles have a significant modifying effect on its surface morphology.

It is well known that the higher the temperature during annealing, the more easily the nanoparticles are agglomerated [50]. As shown in Fig. 1b3, c3, the MoSe_2_ nanoparticles in MoSe_2_/PNC-60 are significantly larger in size and exhibit a significant tendency to agglomerate compared to MoSe_2_/MoO_2_/PNC-60. Surprisingly, it can be seen from the TEM image of MoSe_2_/MoC/PNC-60 (Fig. 1d3) that its MoSe_2_ nanoparticles become smaller in size, more dispersed in distribution, and more uniform in both size and degree of dispersion. This can prove that the MoC interlayer has an immobilizing effect on MoSe_2_ nanoparticles, which can both prevent their agglomeration and inhibit their overgrowth during high-temperature heat treatment. The uniformly distributed MoSe_2_ nanoparticles are more beneficial to the dissipation of EMWs. Their high-resolution TEM (HRTEM) images are shown in Fig. 1b4–d4. In Fig. 1b4, lattice spacing of 0.322 and 0.285 nm, representing to the (004) and (100) crystal planes of MoSe_2_, can be observed from where the carbon and MoSe_2_ nanoparticles adjoined. There are also 0.281 and 0.243 nm lattice spacing, corresponding to the (− 102) and (− 211) crystal planes of MoO_2_. The fast Fourier transform (FFT) corresponding to the two lattices has been given in the inset, demonstrating the generation of MoSe_2_ and MoO_2_ heterostructures (MoSe_2_/MoO_2_) and that MoSe_2_/MoO_2_/PNC-60 is incompletely selenated. And in the HRTEM image of MoSe_2_/PNC-60 (Fig. 1c4), only the lattice belonging to MoSe_2_ can be observed at the junctions of carbon and molybdenum selenide nanoparticles. The lattice spacing of 0.324 and 0.736 nm, respectively, corresponding to the (004) and (002) crystal planes, proves that MoSe_2_/PNC-60 is fully selenated. Significantly, in the HRTEM image of MoSe_2_/MoC/PNC-60 (Fig. 1d4), in addition to the corresponding lattice of MoSe_2_, 0.254 and 0.276 nm crystal plane spacing belonging to MoC can be observed, representing to the (100) and (001) crystal planes, respectively. And its FFT inset also demonstrates the generation of MoSe_2_ and MoC heterostructures (MoSe_2_/MoC). Figure 1b5–d5 provides the electron diffraction patterns of the three samples. In addition to a series of typical diffraction rings of MoSe_2_, the (− 212) crystal plane belonging to MoO_2_ and (101) crystal plane belonging to MoC are also observed in Fig. 1b5, d5, respectively, further demonstrating the successful synthesis of the three composites.

**Fig. 2:**
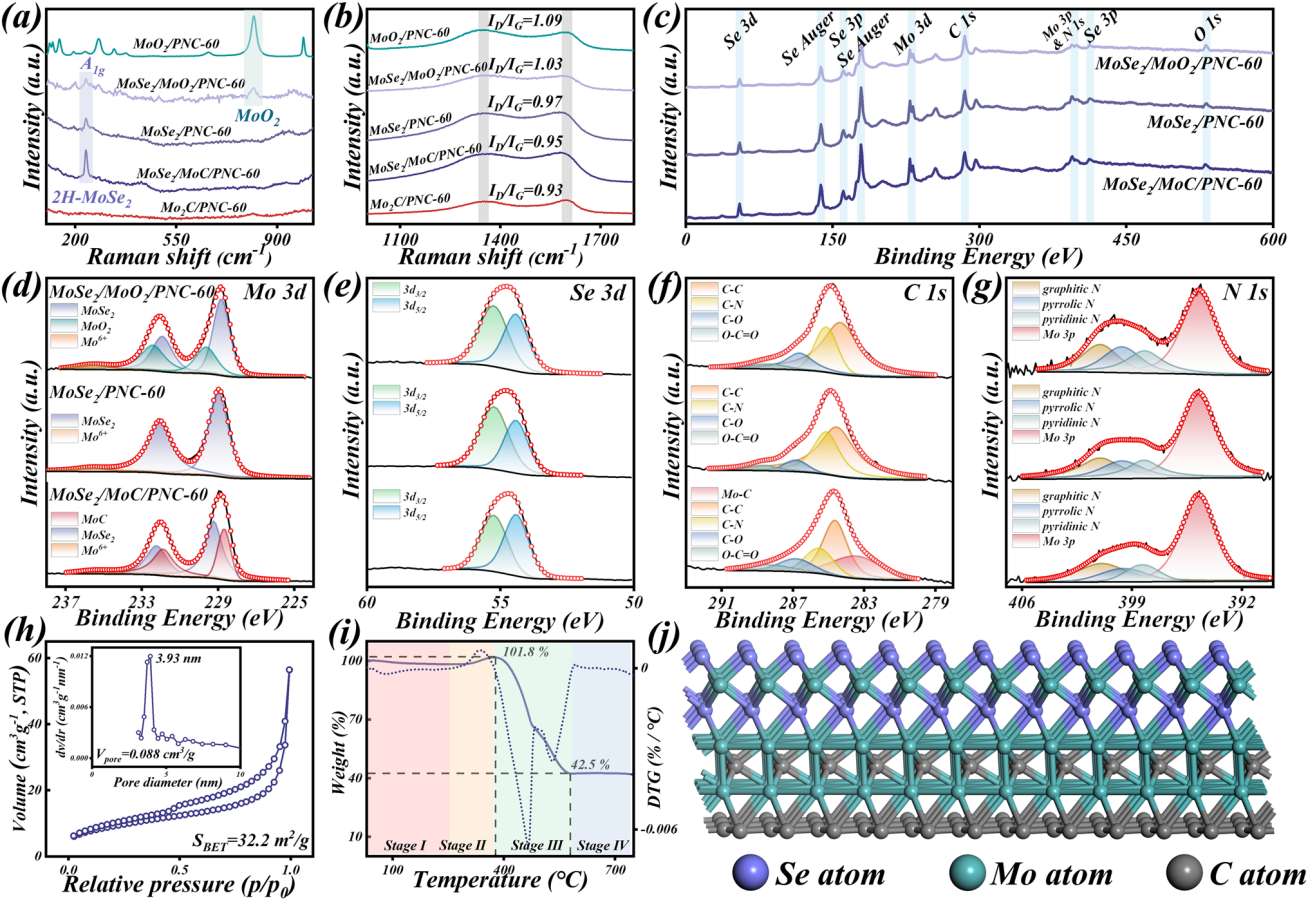
**a, b** Raman spectra of each sample (*x* = 60), **c–g** XPS spectra of MoSe_2_/MoO_2_/PNC-60, MoSe_2_/PNC-60, and MoSe_2_/MoC/PNC-60, **h** N_2_ adsorption–desorption isotherms and **i** TGA and DTG of MoSe_2_/MoC/PNC-60

The porous characteristic can be further illustrated from the N_2_ adsorption–desorption isotherms of MoSe_2_/MoC/PNC-60 (Fig. 2h). The hysteresis loop in the high pressure region indicates the presence of abundant mesopores. In addition, the specific surface area (*S*_BET_) calculated according to the Brunauer–Emmett–Teller (BET) method and the major pore size and total pore volume (*V*_pore_) calculated according to the BJH theory are also presented in the figure. The three-dimensional conductive network structure formed by the porous structure of PNC can greatly enhance the electron transport performance and contribute to the enhancement of the conductive loss mechanism of EMW absorption. The TG and DTG analysis of MoSe_2_/MoC/PNC-60 (Fig. 2i) is divided into four main stages. First, a slight weight loss can be observed with the increase in temperature, caused by the vaporization of water in air atmosphere adsorbed by the sample as the temperature increases. After the temperature reaches 260 °C, MoSe_2_ is oxidized to MoO_3_ and SeO_2_, and an increase in the sample mass can be observed. After about 370 °C, it comes to the third stage, which is also a weight loss stage. According to the two distinct peaks of the DTG curve, it is possible to demonstrate the presence of two types of material weight loss, respectively, sublimation of SeO_2_ and the oxidation of the carbon matrix [51]. Finally, after the temperature comes to 580 °C, the weight remains almost constant, leaving only the presence of MoO_3_ [52]. The Raman spectra of each sample are shown in Fig. 2a, b. In the Raman patterns of the MoO_2_/PNC-60 and MoSe_2_/MoO_2_/PNC-60 samples, the Raman peak at 820 cm^−1^ represents the presence of MoO_2_ [53]. Moreover, in the Raman patterns of the three hierarchical porous molybdenum selenide samples, there is a distinct Raman peak at 238 cm^−1^ attributable to the out-of-plane mode (A_1g_) of MoSe_2_, which represents that the generated MoSe_2_ nanoparticles are in the 2H phase (2H-MoSe_2_) [54]. It further illustrates the successful synthesis of hierarchical porous MoSe_2_ with different components. Furthermore, it is evident that all samples have peaks near 1350 and 1600 cm^−1^, which can be attributed to the D peak signifying the disordered carbon structure and the G peak signifying the graphitized carbon structure, respectively. Crucially, the ratio of the intensity of D peak to G peak (*I*_D_/*I*_G_) is a reflection of the degree of carbon graphitization [55]. As can be seen, the *I*_D_/*I*_G_ values for each sample show a tendency to depending on the increase in heat treatment temperature. However, they both have values around 1, indicating that about half of the carbon is graphitized and the degree of graphitization is relatively similar.

For a more accurate and in-depth analysis of the elemental components and chemical status of the hierarchical porous molybdenum selenide surface, the sample was characterized by XPS. The characteristic peaks belonging to Mo, Se, C, N, and O elements as well as the auger peak of Se can be easily observed in its corresponding XPS total spectrum (Fig. 2c), which proves the successful synthesis of the sample [39]. The Mo 3*d* patterns of the three samples (Fig. 2d) differ in their decomposition forms. Specifically, the Mo 3*d* pattern of MoSe_2_/MoO_2_/PNC-60 can be decomposed into five peaks, with peaks located at 228.8 and 231.9 eV corresponding to MoSe_2_ and peaks located at 229.6 and 232.4 eV corresponding to MoO_2_ [56]. Furthermore, Mo 3*d* of MoSe_2_/PNC-60 can be decomposed into three peaks, with peaks located at 229.0 and 232.1 eV attributed to MoSe_2_ [57]. Finally, MoSe_2_/MoC/PNC-60 can also be decomposed into five peaks, where the peaks at 228.6 and 231.8 eV correspond to MoC, while the peaks at 229.2 and 232.2 eV can be assigned to MoSe_2_ [42, 58]. Also, the micropeaks in three samples near 235.6 eV attributed to the presence of small amounts of Mo^6+^ are due to oxidation during XPS tests in air [39]. The Se 3*d* patterns of the three (Fig. 2e) can be decomposed into two peaks, Se^2−^ 3*d*_5/2_ at 54.4 eV and Se^2−^ 3*d*_3/2_ at 55.2 eV, respectively [59]. In the corresponding C 1*s* patterns (Fig. 2f), all samples can decompose peaks near 284.5 eV (C–C), 285.3 eV (C–N), 286.8 eV (C–O), 288.5 eV (O–C=O), and more remarkably, only MoSe_2_/MoC/PNC-60 can decompose peaks belonging to Mo-C at 283.4 eV. Nitrogen-rich PVP introduces N atoms in the carbon matrix when carbonized at high temperatures, as evidenced by the corresponding N 1*s* patterns (Fig. 2g). The N 1*s* patterns of all samples can be decomposed into four peaks, namely Mo 3*p*, pyridine N, pyrrole N, and graphitized N located at 394.8, 398.2, 399.6, and 400.9 eV, respectively [60]. Multispecies N atom doping can enhance the absorption of EMWs in several ways [61]. The O 1*s* patterns (Fig. S12) can be decomposed into two peaks, the peaks at 530.8 and 532.7 eV are attributed to lattice oxygen (*O*_L_) and adsorbed oxygen (*O*_A_), respectively. All samples carry *O*_L_ due to inevitable surface oxidation in air. The *O*_A_ and *O*_L_ ratios were calculated, apparently MoSe_2_/MoO_2_/PNC-60 has a higher *O*_L_ content due to the MoO_2_ heterostructure. By XPS analysis, we further determined the rationality of the synthesis strategy and, more importantly, demonstrated the formation of Mo-C bonds. This implies that after the carbonization of PVP, MoC is grown in situ on PNC. Combined with the formation of MoSe_2_/MoC heterogeneous structure confirmed by the previous TEM characterization, it can be reasonably inferred that a small amount of MoC acts as an intermediate layer in MoSe_2_/MoC/PNC-60, connecting MoSe_2_ nanoparticles with PNC to form a MoSe_2_-MoC-C multiple heterogeneous interfacial structure (as shown in Fig. 2j).

### EMW Absorption Performance

The sample powder prepared was homogeneously mixture with paraffin wax (sample powder weight ratio of 27.5 wt%) in order to investigate the EMW attenuation properties of the sample. Two important parameters: the complex permittivity (*ε*_*r*_ = *ε′–jε″*) and the complex permeability (*μ*_*r*_ = *μ′–jμ″*) can be measurable with vector network analyzer, and they are of key importance to determine the EMW absorption performance of the material. The real and imaginary parts of the complex permittivity (*ε′* and *ε″*) represent the storage and consumption ability for electrical energy, respectively. The real and imaginary parts of the complex permeability (*μ'* and *μ"*) are used to describe the stored and consumed capacity for magnetic energy, respectively [62, 63]. As the prepared samples are non-magnetic, the research on magnetic loss can be ignored in this work.

Impedance matching is the primary principal to be considered when devising a high-performance absorber. Simply put, when the EMW propagates from the air to the absorbers surface, the impedance of the absorber should be approaching to the impedance of the air. At this time, the EMW tend to enter internal of the absorber rather than being reflected, that is, the impedance match, otherwise it is impedance mismatch. The impedance values can be deduced as follows [64, 65]:

3 $$Z = \frac{{Z_{{{\text{in}}}} }}{{Z_{0} }} = \sqrt {\frac{{\mu_{r} }}{{\varepsilon_{r} }}} \tanh \left( {j\frac{2\pi fd}{c}\sqrt {\varepsilon_{r} \mu_{r} } } \right)$$

The *Z* value is related to frequency (*f*) and thickness (d). When the Z value approaching 1, which means that the impedance matching of the absorber is good at this time, and the EMW can enter the absorber, so as to carry out the energy absorption and conversion.

**Fig. 3:**
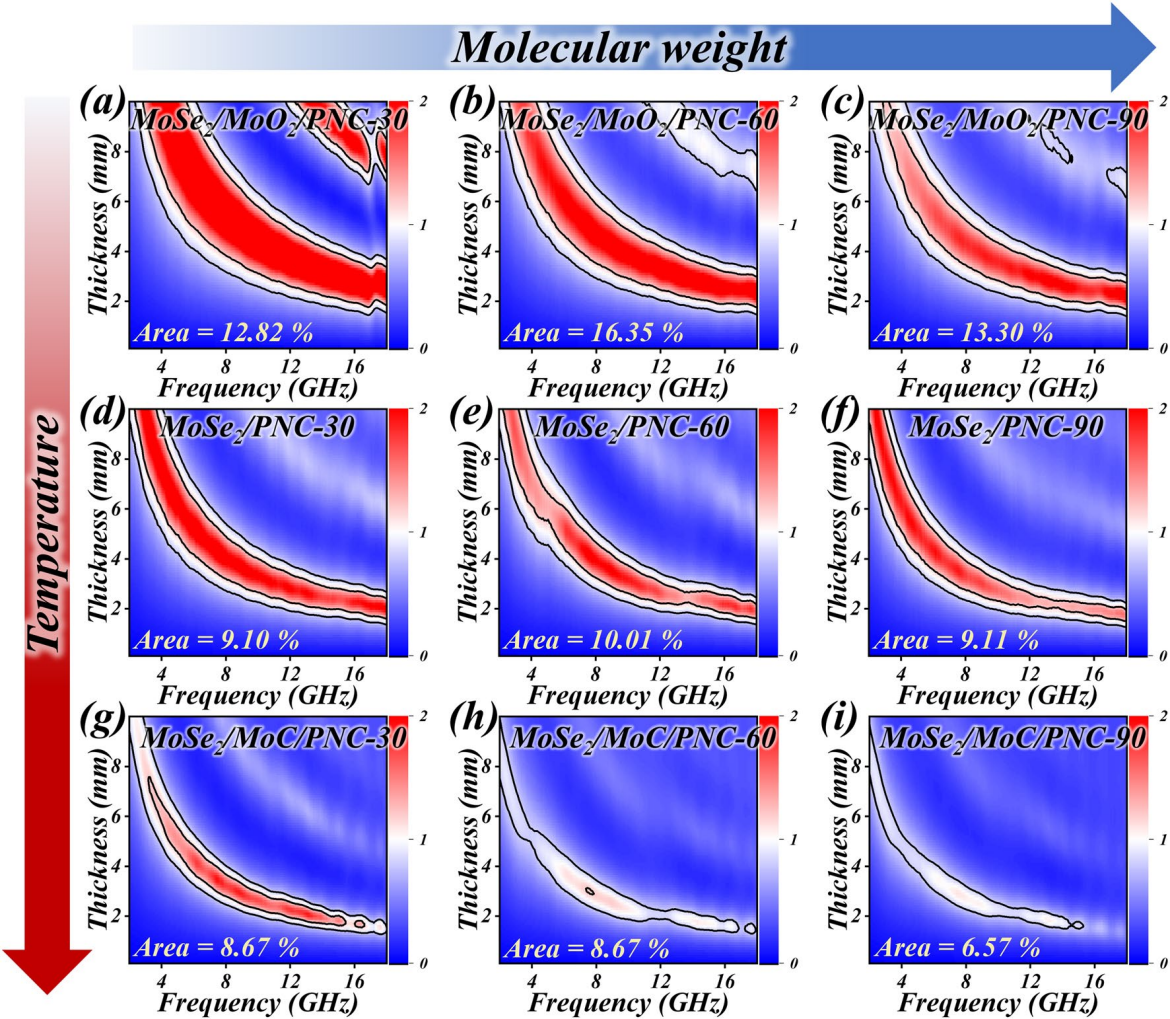
normalized input impendence Z of **a–c** MoSe_2_/MoO_2_/PNC-x, **d–f** MoSe_2_/PNC-x, and **g–i** MoSe_2_/MoC/PNC-x

As mentioned above, hierarchical porous molybdenum selenide with different structures was prepared by using different species of PVP. In order to nuance this study, the impedance matching characteristics of hierarchical porous molybdenum selenide with different structures were first investigated. Figure 3 indicates the 2D plots of impedance matching performance of MoSe_2_/MoO_2_/PNC-x, MoSe_2_/PNC-x, and MoSe_2_/MoC/PNC-x, respectively. The white region between *Z* = 0.8 and *Z* = 1.2 is marked with black lines, the larger the region circled, the better the impedance matching performance. It can be evidently observed that the impedance matching of the final prepared samples using PVP-60 as the carbon source is the most superior when the components of the hierarchical porous molybdenum selenide are the same (in MoSe_2_/MoC/PNC-x, the regions have the same area when *x* = 30 and 60).

**Fig. 4:**
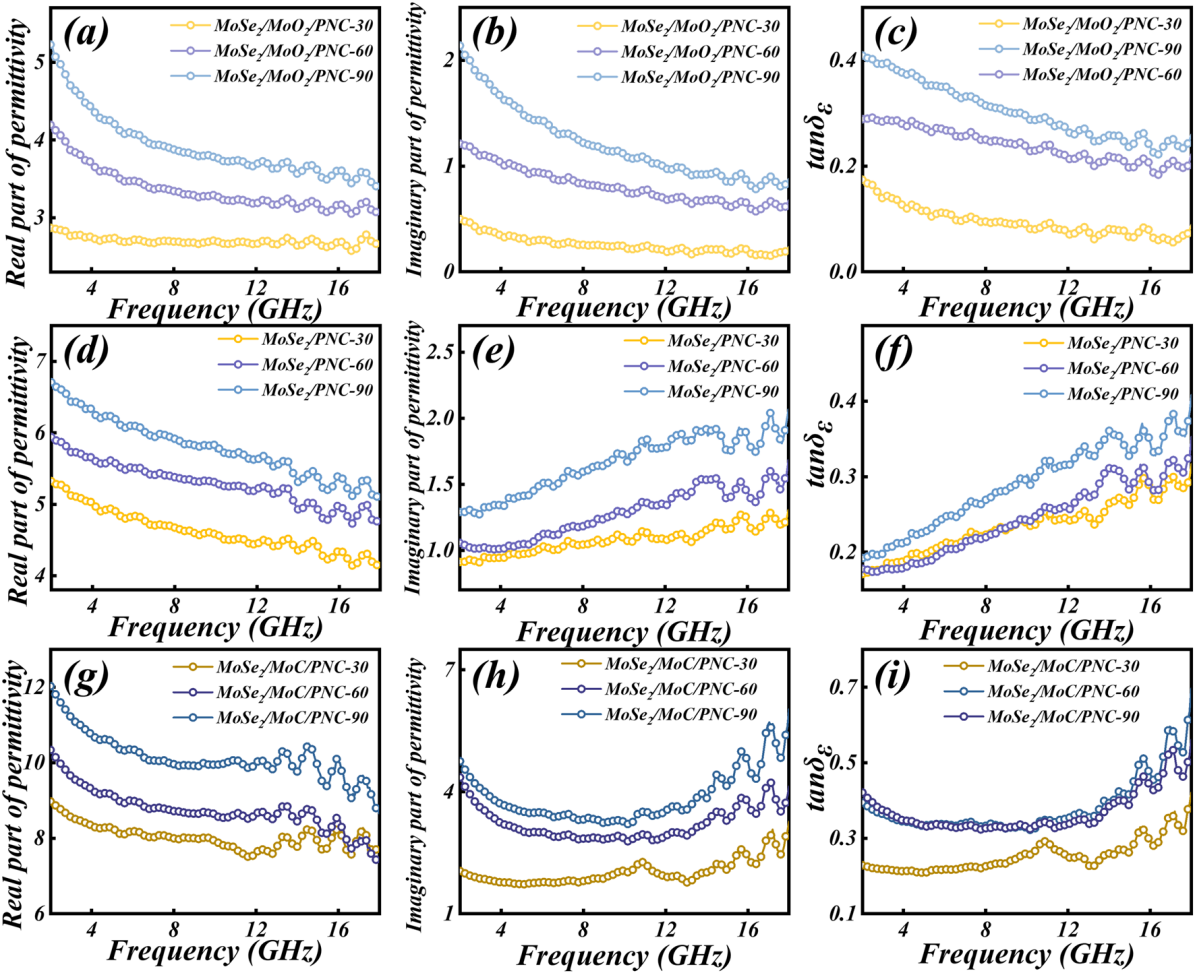
Real part of permittivity constant, imaginary part of permittivity constant, and tangent of permittivity constant of **a–c** MoSe_2_/MoO_2_/PNC-x, **d–f** MoSe_2_/PNC-x, and **g–i** MoSe_2_/MoC/PNC-x

The trend of the complex permittivity parameters (Fig. 4) tends to be consistent for the different structures of hierarchical porous molybdenum selenide as the composition changes. According to the previous SEM results, since the PNC skeleton formed with PVP-K90 as the carbon source is thinner and has a denser distribution of holes, this may provide more abundant conductive paths and thus enhance the dielectric loss of the material [66]. And too high permittivity parameters can cause impedance mismatch in EMW absorption [67]. This explains the poor impedance matching performance of the sample with stronger dielectric loss capability (*x* = 90). And when *x* = 60, the stronger dielectric loss coexists with the better impedance matching property, strongly demonstrating the optimization of the impedance matching property by this structure. Therefore, a more in-depth study of the EMW absorption performance of the sample with *x* = 60 is chosen subsequently.

**Fig. 5:**
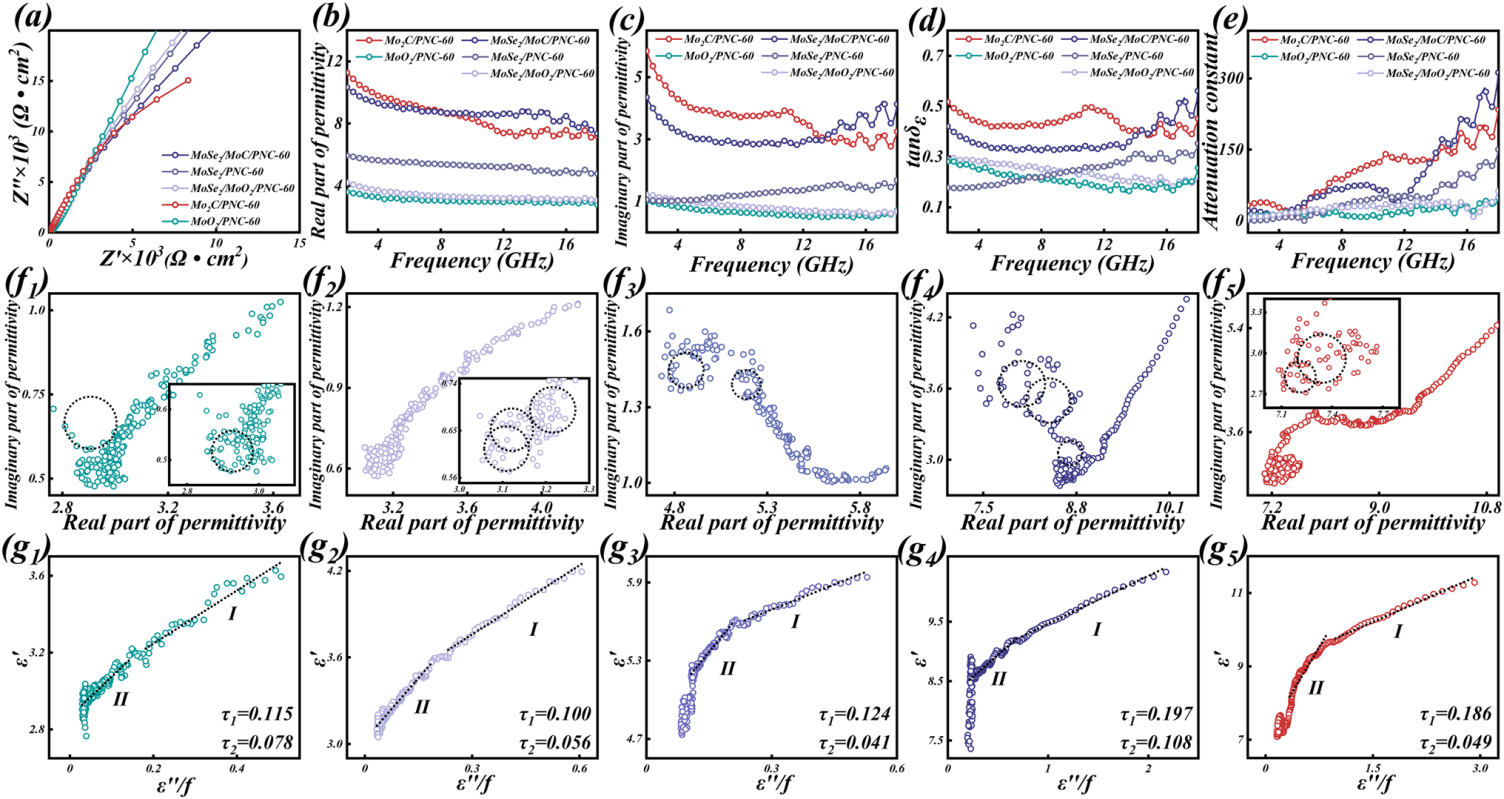
Electrochemical characterization in 3.5 wt% NaCl solution and electromagnetic parameters. **a** Nyquist plots, **b** Real part of permittivity constant, **c** imaginary part of permittivity constant, **d** tangent of permittivity constant, **e** attenuation constant of each sample (*x* = 60). Cole–Cole plots (**f**_**1**_**–f**_**5**_) and the relationship between *ε′* and *ε″/f* (**g**_**1**_**–g**_**5**_) of MoO_2_/PNC-60, MoSe_2_/MoO_2_/PNC-60, MoSe_2_/PNC-60, MoSe_2_/MoC/PNC-60 and Mo_2_C/PNC-60

According to a previous study, MoC can promote ion and electron transport as well as structural stability in composites [42]. This can also be demonstrated by electrochemical tests performed with a three-electrode system in a 3.5 wt% NaCl solution simulating a marine environment. From the Nyquist plots of each sample (Fig. 5a), it can be seen that MoSe_2_/MoC/PNC-60 has a smaller capacitive arc than MoSe_2_/PNC-60, indicating that it has a smaller impedance and is more conductive [68].

When x = 60, the values of *ε′*, *ε″*, dielectric loss factor (tan*δ*_*ε*_), and average tan*δ*_*ε*_ for each sample with different components are shown in Fig. 5b–e, and it can be clearly seen that Mo_2_C/PNC-60 has the most superior complex permittivity parameters. According to the results of the previous Nyquist plots, this may be attributed to the high conduction loss due to its higher conductivity. It is noteworthy that the conduction loss and interfacial polarization are simultaneously enhanced due to the MoC that both enhances the conductivity of MoSe_2_/MoC/PNC-60 and introduces multiple heterogeneous interfaces. Therefore, its complex permittivity parameters are significantly superior to those of MoSe_2_/PNC-60. In addition, although the conductivity of MoSe_2_/MoO_2_/PNC-60 is weaker than that of MoSe_2_/PNC-60, it has enhanced dielectric loss by virtue of the interfacial polarization introduced by the MoSe_2_/MoO_2_ heterostructure, giving it a similar level of complex permittivity parameters as MoSe_2_/PNC-60 (Fig. S13).

In order to further reveal the dielectric loss mechanism, the Debye theory is introduced here to describe the polarization relaxation behavior, which is given by [69]:

4 $$\varepsilon^{{\prime }} = \varepsilon_{\infty } + \frac{{\varepsilon_{s} - \varepsilon_{\infty } }}{{1 + (2\pi f)^{2} r^{2} }}$$

5 $$\varepsilon^{{\prime \prime }} = \frac{{\omega \tau (\varepsilon_{s} - \varepsilon_{\infty } )}}{{1 + (2\pi f)^{2} r^{2} }}$$

where *ε*_s_ is the static dielectric constant, *ε*_*∞*_ is the optical dielectric constant, *f* is the frequency, and *t* is the polarization relaxation time. The Cole–Cole formula is expressed by this equation [70, 71]:

6 $$\left( {\varepsilon^{{\prime }} - \frac{{\varepsilon_{s} + \varepsilon_{\infty } }}{2}} \right)^{2} + (\varepsilon^{{\prime \prime }} )^{2} = \left( {\frac{{\varepsilon_{s} - \varepsilon_{\infty } }}{2}} \right)^{2}$$

If the sample suffers a polarization relaxation process, then curves plotted from *ε′* and *ε″* will shape a semicircle, with each semicircle representing a Debye relaxation process. The Cole–Cole curves of each sample (Fig. 5f1–f5) clearly indicate multiple distorted semicircular shapes, which indicates the presence of additional polarization relaxation processes. On the one hand, the defective carbon and N atom doping on the PNC leads to the generation of defective polarization. On the other hand, multiple interfacial polarizations are introduced for the material between the porous structure and the air medium, as well as between different components (MoSe_2_ and PNC, MoSe_2_ and MoO_2_, MoSe_2_ and MoC, etc.). Apparently, the Cole–Cole curves of MoSe_2_/MoO_2_/PNC-60 and MoSe_2_/MoC/PNC-60 show more semicircular shapes due to the presence of additional heterogeneous interfaces in them. This proves that the interface engineering brings more interfacial polarization relaxation process for them, which contributes to enhance their dielectric loss capability and improve the attenuation ability to EMWs.

The correlation between *ε′* and *ε″/f* also allows further proof of the polarization behavior of the sample. According to Eqs. (4) and (5), the following equations result [72, 73]:

7 $$\varepsilon^{{\prime }} = \frac{1}{2\pi \tau }\frac{{\varepsilon^{{\prime \prime }} }}{f} + \varepsilon_{\infty }$$

If polarization relaxation presence in dielectric loss, *ε′* and *ε″/f* will linearly correlate and the slope is available to calculate the polarization relaxation time [74]. A linear fit reveals that the relationship between *ε′* and *ε″/f* curves for each sample are fitted as two straight lines with different slopes (Fig. 5g1, g2). This result further proves the presence of multiple polarization processes (defect polarization and interfacial polarization) for each sample [75].

The attenuation constant (*α*) is a vital element to be aware of when designing a high-performance absorber, which represents the capability of the absorber to convert EMW energy into other energy, meaning the ability to absorb and attenuate EMWs. *α* can be deduced from the next equation [76, 77]:

8 $$\alpha = \frac{\sqrt 2 \pi f}{c}\sqrt {(\mu^{\prime \prime } \varepsilon^{\prime \prime } - \mu^{\prime } \varepsilon^{\prime } ) + \sqrt {(\mu^{\prime \prime } \varepsilon^{\prime \prime } - \mu^{\prime } \varepsilon^{\prime } )^{2} + (\mu^{\prime } \varepsilon^{\prime \prime } + \mu^{\prime \prime } \varepsilon^{\prime } )^{2} } }$$

The *α* curves of each sample are shown in Fig. 5e, and it is evident that the attenuation ability of MoSe_2_/MoC/PNC-60 far exceeds that of MoSe_2_/PNC-60, while reaching a high loss level similar to that of Mo_2_C/PNC-60. In addition, the attenuation ability of MoSe_2_/MoO_2_/PNC-60 is similar to that of MoSe_2_/PNC-60. This is coherent with the results of the previous findings on dielectric loss and further demonstrates the importance of the interfacial polarization induced by the heterogeneous interface for enhancing the EMW absorption of the material.

**Fig. 6:**
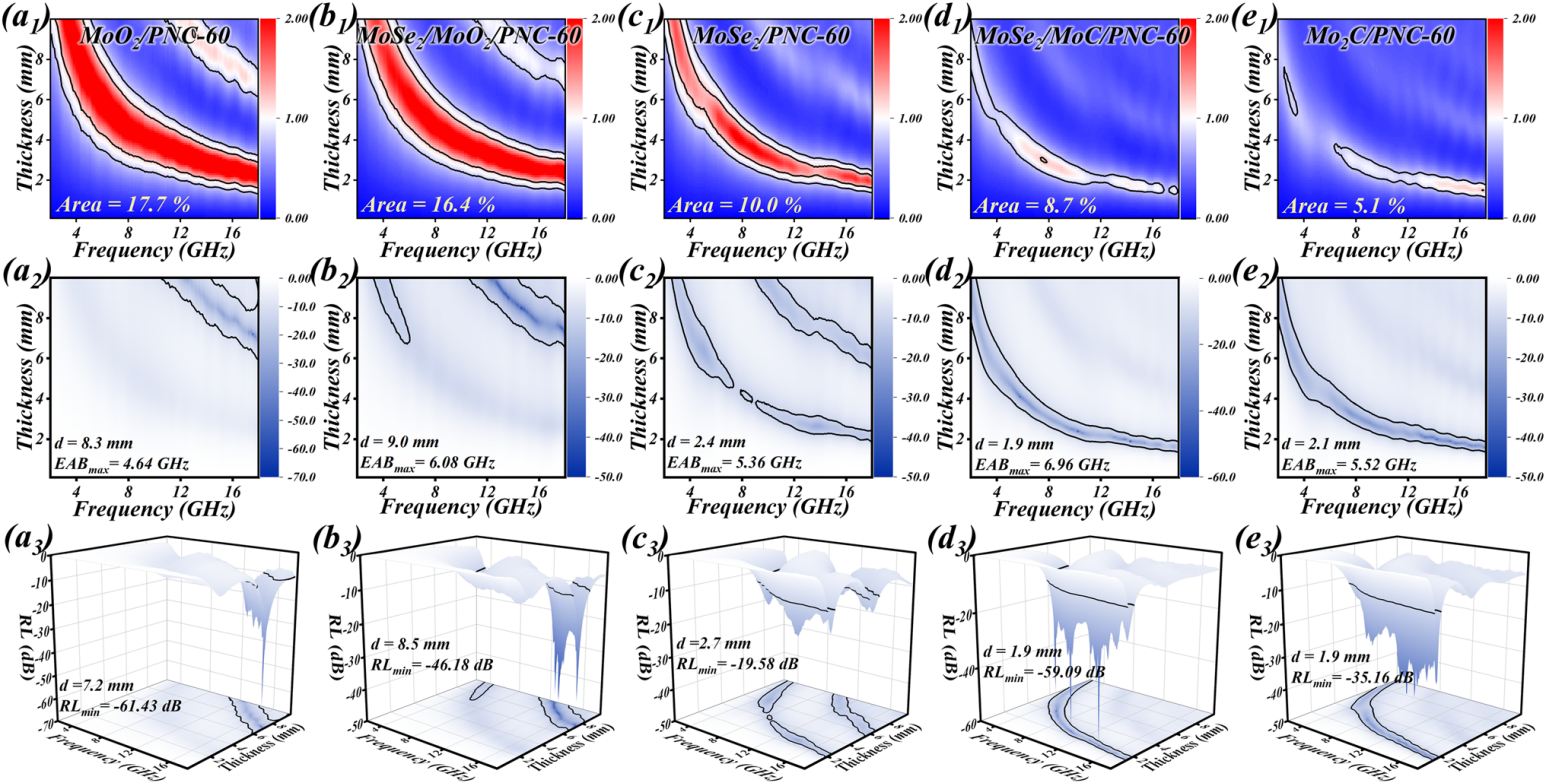
The normalized input impendence Z (**a**_**1**_**–e**_**1**_), 2D RL (**a**_**2**_**–e**_**2**_) and 3D RL (**a**_**3**_**–e**_**3**_) images of each sample (*x* = 60)

**Fig. 7:**
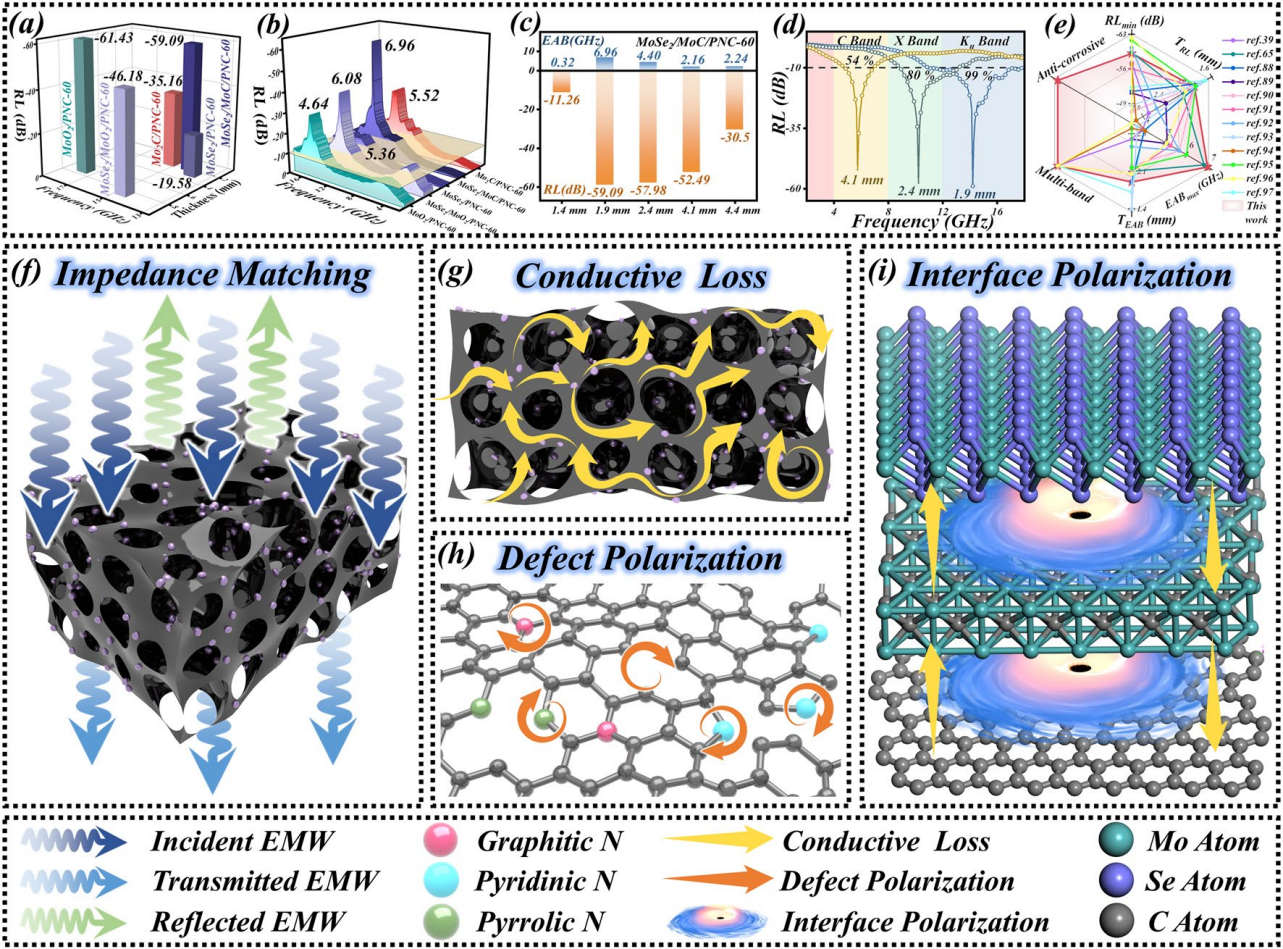
The comparison of each sample (*x* = 60) **a** Rl_min_ and **b** EAB_max_ , EMW absorption performance of MoSe_2_/MoC/PNC-60 in **c** different thickness and **d** different frequency bands. **e** Comparison of MoSe_2_/MoC/PNC-60 and other works, **f–i** EMW absorption mechanism

Figure 6a1–e1 indicates the corresponding impedance matching plots. It can be seen that the impedance matching is weakened with the increase in dielectric performance. With similar structures, this coincides with the relationship between complex permittivity parameters and impedance matching as mentioned before. In order to assess more intuitively the EMW absorption performance of the absorber, the RL value and EAB are calculated according to the line transmission theory (Eqs. (1) and (2)). Generally speaking, the absorption of incident EMWs is up to 90% for RL < − 10 dB. The range of frequencies at which this requirement is achieved at a certain thickness is the EAB. In Fig. 6a2–e2 2D RL plots and Fig. 6a3–e3 3D RL plots, this region is marked with black lines. As can be seen that the EMW absorption performance of MoSe_2_/MoC/PNC-60 is the most superior among a group of samples, especially showing an RL_min_ of − 59.09 dB and an EAB_max_ of 6.96 GHz at 1.9 mm. Figure 7a, b indicates the visualized comparison of RL_min_ and EAB_max_ for each sample, respectively (the yellow plane in the figure represents the plane with RL = − 10 dB). It can be seen that MoSe_2_/MoC/PNC-60 can obtain lower RL and wider EAB at thinner thicknesses than other samples, and its EMW absorption behavior shows a trend toward lower frequency. From the previous section, although the overall impedance matching performance of MoSe_2_/MoC/PNC-60 is relatively average, it benefits from the continuous impedance matching region at thin thickness and the strong dielectric loss performance brought by the MoC interlayer, which creates the characteristics of thin thickness, strong absorption, and wide frequency band. Additionally, RL performance images at *x* = 30 and 90 (Figs. S14 and S15) obviously demonstrate that they have difficulty satisfying the multifaceted EMW absorption characteristics index.

Notably, three strong RL peaks of MoSe_2_/MoC/PNC-60 were observed in Fig. 6d3, and this was used as a focus for further analysis of the absorption behavior of MoSe_2_/MoC/PNC-60 at different thicknesses. Surprisingly, besides at 1.9 mm, strong RLs of − 57.98 and − 54.49 dB are also obtained at two relatively thin thicknesses of 2.4 and 4.1 mm, respectively, with corresponding EABs of 4.4 and 2.08 GHz (Fig. 7c). The corresponding RL curves at these three thicknesses are shown in Fig. 7d. At a thickness of 1.9 mm, the EAB covers almost the entire *K*_u_-band (12–18 GHz). At a thickness of 2.4 mm, the EAB covers 80% of the X-band (8–12 GHz). At a thickness of 4.1 mm, the EAB covers 54% of the C-band (4–8 GHz). The superior multiband performance can be easily achieved by simply adjusting the thickness in a thin range, showing satisfactory band tunability.

Furthermore, in order to reveal the EMW absorption behavior more deeply, the quarter wavelength matching theory is employed to further investigate the relationship among the matching thickness of MoSe_2_/MoC/PNC-60 and the reflection loss and frequency. Its equation is as follows [78, 79]:

9 $$t_{{\text{m}}} = \frac{n\lambda }{4} = \frac{nc}{{4f_{{\text{m}}} \sqrt {\left| {\mu_{{\text{r}}} } \right|\left| {\varepsilon_{{\text{r}}} } \right|} }}\quad ( = 1,3,5 \ldots )$$

where *t*_m_ is the thickness of the match, *c* is the velocity of the EMW in vacuum, *f*_m_ is the frequency of the match, and |*ε*_r_| and |*μ*_r_| are the modulus of *ε*_r_ and *μ*_r_, respectively. As soon as the phase difference from the reflected and absorbed EMW is 180° (*π*/2), *t*_m_ and *f*_m_ fulfill the above equation and the two EMWs offset each other. At this time, the RL of the absorber will reach the minimum, meaning RL_min_. According to Fig. S16, RL gradually moves toward lower frequencies as the thickness grows. As the results indicate, the RL attains its minimum value at 14.24 GHz when the thickness of the sample is the same as Eq. (8). The experimental results are coherent with the simulated results of the *t*_m_–*f*_m_ curves, proving that the quarter wavelength matching model can precisely describe the behavior of the absorber. More significantly, it can be found that impedance matching (*Z* = 1) is achieved at 1.9, 2.4, and 4.1 mm thicknesses. Combined with the above study, this explains to some extent why MoSe_2_/MoC/PNC-60 can achieve excellent EMW absorption in multiple frequency bands and further demonstrates the extent to which impedance matching is critical for the absorber.

Typically, dielectric loss mechanisms include and originate from conduction loss, interfacial polarization, defect-induced polarization, etc. As the current transmits along the absorber, the intrinsic resistance generates Joule heat, which consumes the energy of the EMW. Additionally, defect sites in the absorber capture the carriers generated in the external alternating electromagnetic field, leading to the propagation of negative carriers, and ultimately the occurrence of defect polarization processes and the associated EMW energy dissipation. Moreover, during alternating electromagnetic fields, owing to the differences in charge retention ability and conductivity of different components, carrier will accumulate at heterogeneous interfaces, evoking intense interfacial polarization and relaxation processes.

Undoubtedly, the loss mechanism of the prepared samples is dominated by dielectric loss, and the included EMW absorption mechanism is illustrated in Fig. 7f–i. First, by changing the carbon precursors, a porous structure with excellent impedance matching properties is achieved, which contributes to the entry of EMWs into the interior of the material, allowing MoSe_2_/MoC/PNC-60 to sufficiently exploit its attenuation capability for EMWs. Then, the porous carbon skeleton constructs a three-dimensional conductive network structure, which results in a rich conduction path that facilitates electron migration and hopping, which dramatically strengthens its conduction loss capability. In addition, the intrinsic defects on the carbon substrate with nitrogen atom doping introduce a huge number of zero-dimensional defects, and these defect sites can trap charge carriers and disrupt the balance of charge distribution, thus causing abundant defect polarization also contributes to the attenuation of EMWs. Finally, the presence of the critical MoC interlayer boosts the conductivity and stability. The rational design of the MoSe_2_–MoC–C heterogeneous interfacial coupling reinforces the conduction loss while introducing multiple interfacial polarizations. With the synergistic effect of multiple mechanisms, the EMW absorption performance of MoSe_2_/MoC/PNC-60 has been comprehensively optimized, especially its absorption performance at multiple frequency bands is particularly remarkable.

### Anticorrosion Property

Applied in the marine environment, the microwave absorber must not only have excellent microwave absorption performance, but also need to have anticorrosion properties. Usually, the anticorrosion ability of coating is assessed by electrochemical measurement techniques using a three-electrode system. The corrosion behavior of different coating was investigated by immersing the working electrodes in seawater solution.

**Fig. 8:**
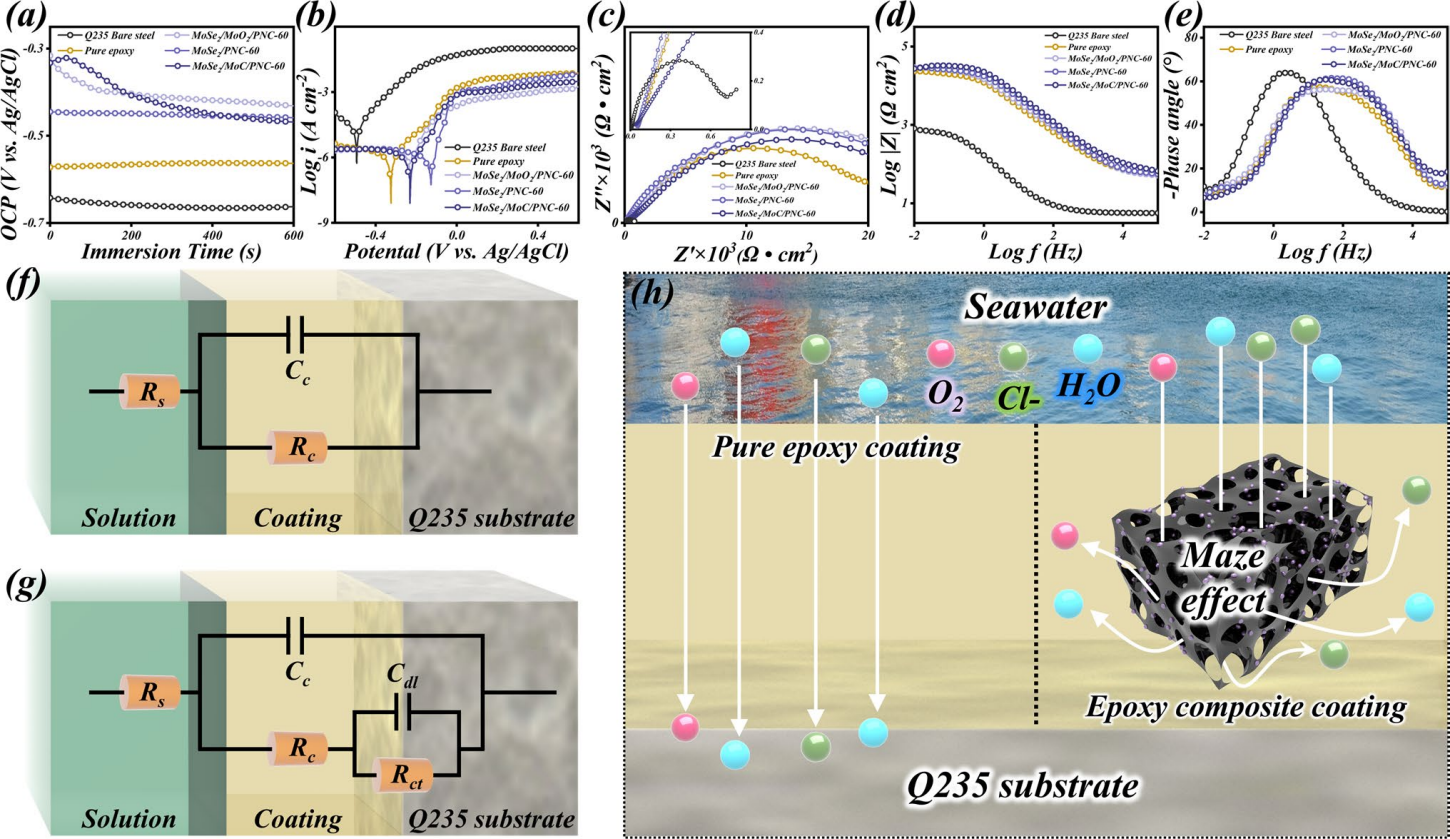
Electrochemical characterization in seawater solution and electromagnetic parameters. **a** OCP curves, **b** polarization kinetic potential curve, **c** Nyquist plots, **d** Bode plots, and **e** phase angle plots of Q235 bare steel, pure epoxy coating, and hierarchical porous molybdenum selenide/epoxy composite coating. **f, g** The equivalent electrical circuit for coating in different stages and **h** schematic of the corrosion protection mechanism

Generally, a higher OCP value represents a lower corrosion trend [80]. The OCP values of each sample (Fig. 8a) gradually stabilized with the measurement time. The Q235 bare steel can be observed to have the lowest OCP values, followed by the pure epoxy coating, indicating that it provides some protection to the bare steel. After introducing hierarchical porous molybdenum selenide as a filler into the epoxy coating, the OCP values were further increased, which indicates that its corrosion resistance was effectively enhanced. Figure 8b indicates the polarization kinetic potential curves of each sample, in which bare steel has the lowest corrosion potential (*E*_corr_), followed by the pure epoxy coating, and all composite coatings have a higher level (Table S2). Furthermore, hierarchical porous molybdenum selenide/epoxy composite coating generally exhibited lower corrosion current density (*I*_corr_). When *E*_corr_ is higher or *I*_corr_ is lower, it means that the sample is more difficult to be oxidized (corroded) and has better corrosion resistance [81]. Figure 8c illustrates the Nyquist curves for bare steel and various coatings. From its inset, it can be observed that the radius of the circle of bare steel is much smaller than that of the other coatings, indicating that it is highly susceptible to corrosion. More significantly, all composite coatings have larger impedance arc than the pure epoxy coating, indicating that the composite coating provides enhanced corrosion protection to bare steel [82]. In the Bode plot, the impedance modulus at 0.01 Hz ($\left| Z \right|_{{0.01\;{\text{Hz}}}}$) can be used as a basis for judging the corrosion resistance [83]. Figure 8d demonstrates that the $\left| Z \right|_{{0.01\;{\text{Hz}}}}$ of the all coatings is much higher than that of bare steel, and the composite coating is slightly higher than the pure epoxy coating, further proving the superior anticorrosion performance of the composite coating. In addition, all coatings have large phase angles as shown in Fig. 8e, it indicates that the coating has typical capacitive properties and can effectively isolate the corrosive medium [84]. Notably, the peak of the phase angle curve for bare steel is closer to the low frequency region (10^–2^–10^0^ Hz) compared to all coatings, which corresponds to the corrosion response of the metal matrix, indicating that corrosion occurred during immersion [85]. This is due to the lack of protection by the coating, and the bare steel is easily corroded in the seawater environment. The above results indicate that the introduced hierarchical porous molybdenum selenide effectively reinforces the anticorrosion performance of the epoxy resin coating.

Figure 8f, g shows the equivalent circuit diagram fitted for the coating during immersion. In the initial stage of coating immersion, the corrosive medium (Cl^−^, H_2_O, O_2_) in seawater does not penetrate into the coating/Q235 substrate interface, and the fitted equivalent circuit is shown in Fig. 8f. And when seawater penetrates the coating and the corrosive medium reaches the surface of the Q235 substrate, the fitted equivalent circuit is shown in Fig. 8g [86]. In the equivalent circuit model, *R*_S_ represents the solution (seawater) resistance, *R*_C_ represents the layer resistance, *C*_C_ represents the coating capacitance, *R*_ct_ represents the charge transfer resistance, and *C*_dl_ represents the double layer capacitance [87]. Figure 8h is a schematic diagram of the corrosion protection mechanism of the composite coating. Normally, due to the existence of more defects and micropores in the pure epoxy coating, affecting the densification of the coating, causing the epoxy coating poor physical barrier properties, corrosive media prone to penetrate into coating via defects, leading to rapid coating deterioration. Pure epoxy coating offered limited protection to the metal substrate, whereas coating filled with hierarchical porous molybdenum selenide exhibited noticeable modification in corrosion protection. On the one hand, carbon skeleton of hierarchical porous molybdenum selenide has a high degree of graphitization after high-temperature heat treatment, which greatly obstructs the electrochemical corrosion reaction. On the other hand, three-dimensional porous structure has abundant tortuous corridors, facilitating the prolongation of diffusion route of corrosive medium and generating “maze effect,” which features reinforce the physical shielding performance of the coating. In summary, hierarchical porous molybdenum selenide chemically and physically robustens the anticorrosion ability of epoxy resin coating.

Compared with other works with porous structure or MoSe_2_ derived materials (Fig. 7e and Table 1), the absorber prepared in this work not only has the characteristics of thin thickness, strong absorption, and wide frequency band, but also, more critically, has multiband tunability and marine corrosion resistance [39, 65, 88–97]. Such comprehensive EMW absorption performance contributes to effective work in a variety of complex electromagnetic environments and is expected to have a broad development after practical application.

**Table 1:** EMW absorption performance of different materials

Sample	RL_min_/dB	*T*_RL_/mm	EAB_max_/GHz	*T*_EAB_/mm	Multiband	Anticorrosion	References
NiCo_2_S_4_@C/PC	− 59.36	2.1	6.8	2.1	None	None	[65]
NiFe_2_S_4_/PC	− 51.41	1.8	4.08	1.9	None	None	[88]
Ni/NiO@PC	− 51.1	2.4	5.12	2.7	Yes	None	[89]
Fe_3_O_4_@FC	− 47.3	1.9	5.68	2.2	None	None	[90]
CeO_2_/PC	− 56.04	1.9	5.28	2.1	None	None	[91]
MoSe_2_	− 60.23	2.56	5.68	2.56	None	None	[92]
CoNi/MoSe_2_	− 48.6	1.8	3.76	1.4	None	None	[93]
MoSe_2_@RGO	− 56.9	8.9	4.12	8.9	Yes	None	[94]
MoS_2_/MoSe_2_	− 61.71	1.88	6.00	2.16	None	None	[95]
MoSe_2_/ZCNF	− 62.30	2.05	5.10	2.05	Yes	None	[96]
MoSe_2_/FeSe_2_ NPs	− 52.26	1.71	4.06	1.71	Yes	None	[97]
Flower-like MoSe_2_	− 57.2	2.7	4	2.7	Yes	None	[39]
MoSe_2_/MoC/PNC-60	− 59.09	1.9	6.96	1.9	Yes	Yes	This work

## Conclusions

In summary, a series of hierarchical porous molybdenum selenide samples with different structures and components were prepared in this work using the SMS strategy. With the optimized impedance matching, the EMW absorption performance of each sample was sufficiently investigated in comparison, and the crucial role played by interface engineering in this work was explored in depth. The results reveal that the impacts of heterogeneous interfaces on the EMW attenuation performance are not negligible. The samples will inherit the characteristics of heterogeneous components to some extent, and more heterogeneous interfaces will induce more interfacial polarization relaxation processes. Therefore, the rational design of interface engineering contributes to optimize the indexes of EMW absorption performance by boosting the dielectric loss. Typically, the samples prepared in this work achieve multiband tunability. Thin thickness, strong absorption, and wide bandwidth EMW absorption characteristics can be obtained in C, X, and K_u_ bands by adjusting the thickness, and additionally have marine corrosion resistance. Such comprehensive EMW absorption performance promises to achieve sufficient exploitation in complex electromagnetic environments. This research provides an important reference and support for the design of multifunctional, multiband absorbers through interfacial engineering.

## Supplementary Information

Below is the link to the electronic supplementary material.

Supplementary file1 (PDF 2045 kb)
