# Supplementary material for: Multiphase Interfacial Regulation Based on Hierarchical Porous Molybdenum Selenide to Build Anticorrosive and Multiband Tailorable Absorbers
Source: Nanomicro Lett. 2023 Nov 6;16:6. doi: 10.1007/s40820-023-01212-4 (PMC10627983; doi:10.1007/s40820-023-01212-4)
Supplement: Supplementary file 1 — Supplementary file1 (PDF 2045 kb) [file 40820_2023_1212_MOESM1_ESM.pdf]

Supporting Information for

Multiphase Interfacial Regulation Based on Hierarchical Porous Molybdenum Selenide to Build Anticorrosive and Multiband Tailorable Absorbers

Tianbao Zhao[1, 2, #], Zirui Jia[3, #, *], Jinkun Liu[2, #], Yan Zhang[2], Guanglei Wu[2, *], Pengfei Yin[1, *]

[1]College of Science, Sichuan Agricultural University, Ya'an 625014, P. R. China

[2]Institute of Materials for Energy and Environment, State Key Laboratory of Bio-fibers and Eco-textiles, College of Materials Science and Engineering, Qingdao University, Qingdao 266071, P. R. China

[3]College of Chemistry and Chemical Engineering, Qingdao University, Shandong, Qingdao 266071, P. R. China

# Tianbao Zhao, Zirui Jia and Jinkun Liu contributed equally to this work.

*Corresponding authors. E-mail: jiazirui@qdu.edu.cn (Z. Jia); wuguanglei@mail.xjtu.edu.cn & wuguanglei@qdu.edu.cn (G. Wu); yinpengfei@sicau.edu.cn (P. Yin)

Supplementary Figures and Tables

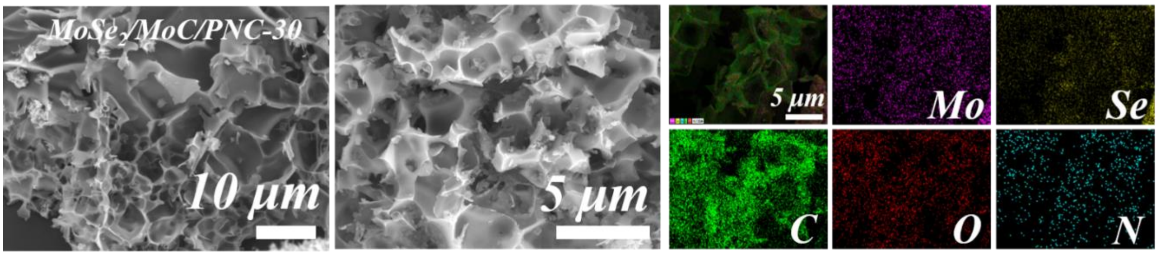

Fig. S1 SEM images and EDS element mapping (Mo, Se, C, N, and O) of $MoSe_2$/MoC/PNC-30

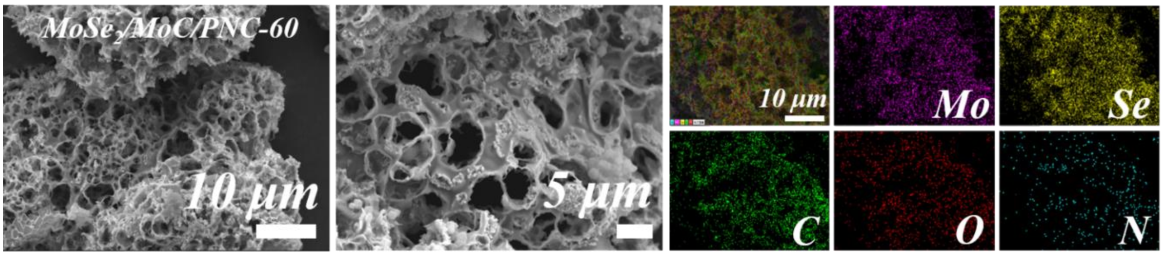

Fig. S2 SEM images and EDS element mapping (Mo, Se, C, N, and O) of $MoSe_2$/MoC/PNC-60

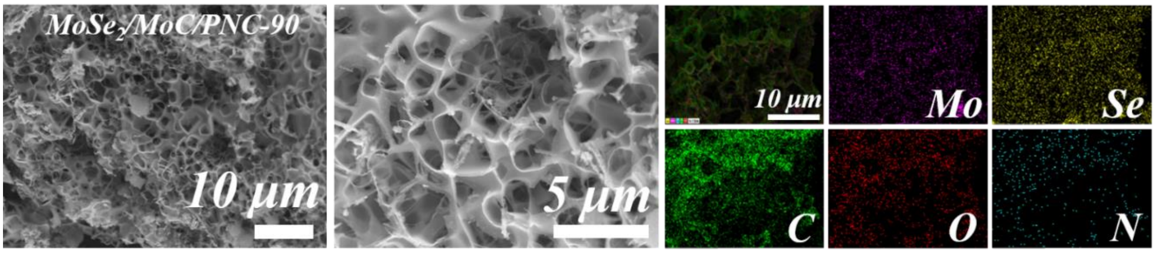

Fig. S3 SEM images and EDS element mapping (Mo, Se, C, N, and O) of $MoSe_2$/MoC/PNC-90

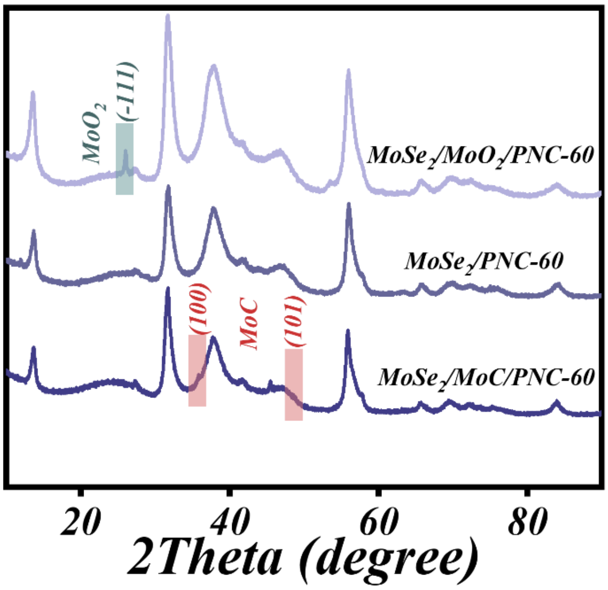

Fig. S4 XRD patterns of $MoSe_2$/MoO_2/PNC-60, $MoSe_2$/PNC-60, and $MoSe_2$/MoC/PNC-60

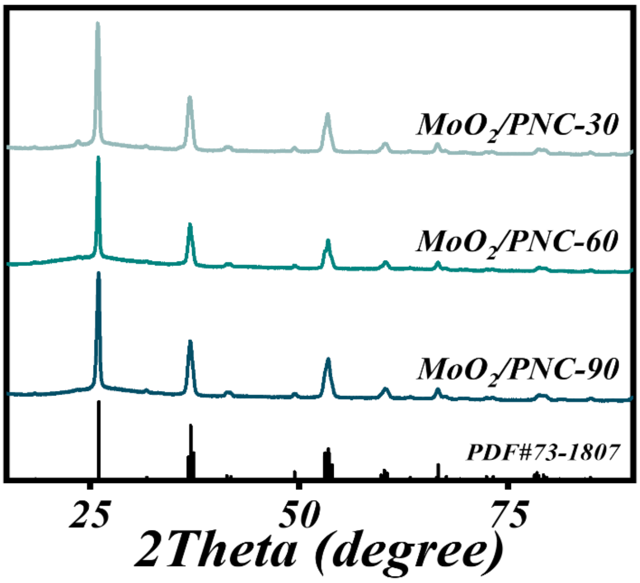

Fig. S5 XRD patterns of MoO_2/PNC-x

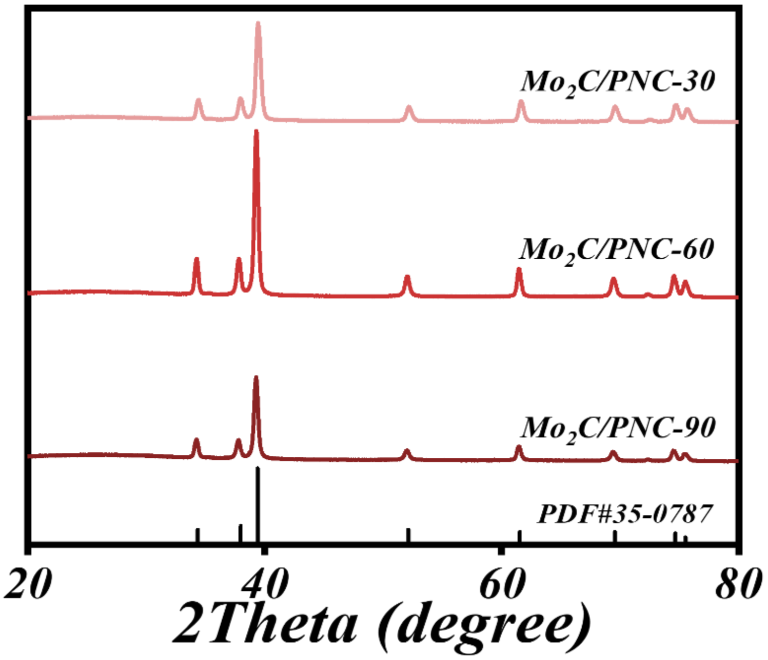

Fig. S6 XRD patterns of Mo_2C/PNC-x

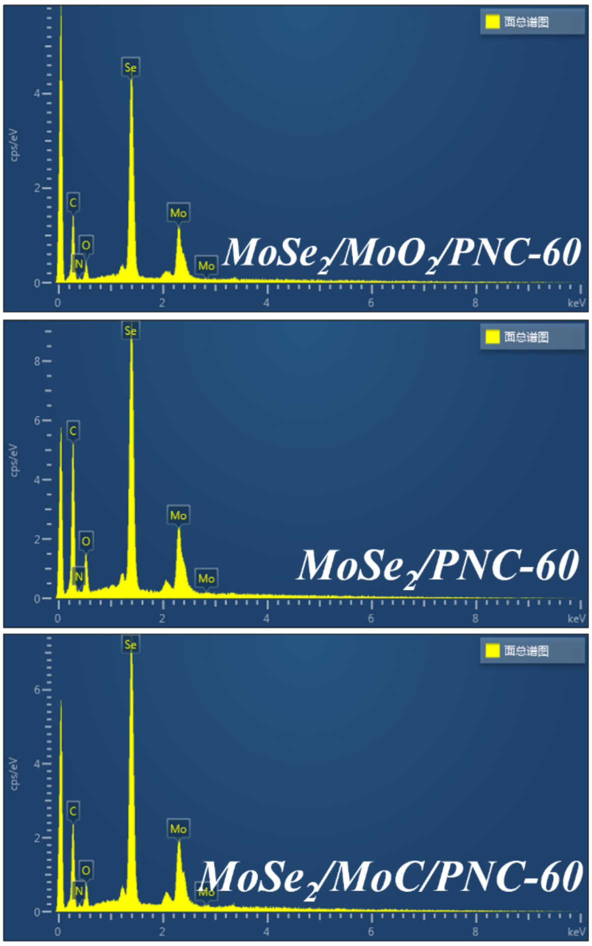

Fig. S7 EDS spectrum of $MoSe_2$/MoO_2/PNC-60, $MoSe_2$/PNC-60, and $MoSe_2$/MoC/PNC-60

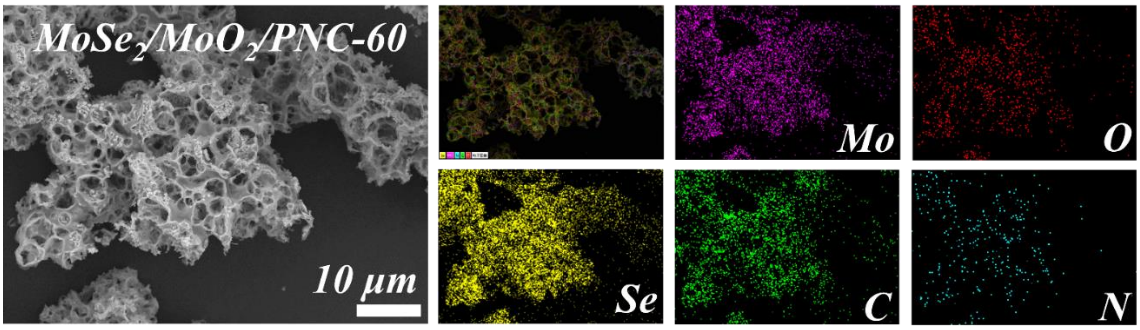

Fig. S8 SEM images and EDS element mapping (Mo, Se, C, N, and O) of $MoSe_2/MoO_2/PNC-60$

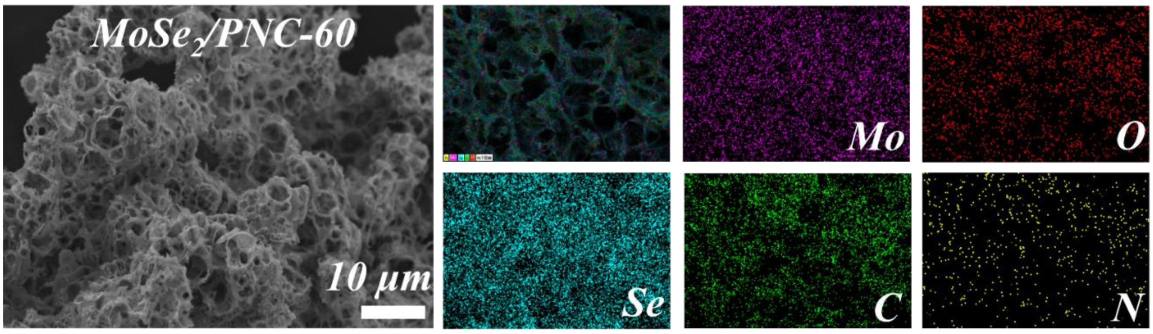

Fig. S9 SEM images and EDS element mapping (Mo, Se, C, N, and O) of $MoSe_2$/PNC-60

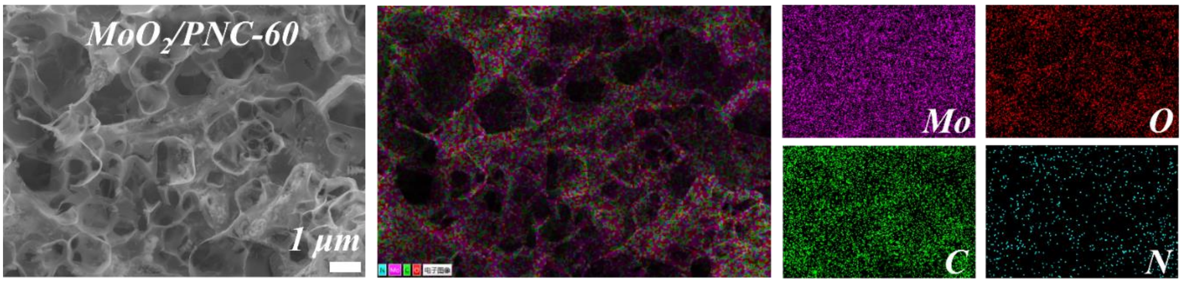

Fig. S10 SEM images and EDS element mapping (Mo, C, N, and O) of MoO_2/PNC-60

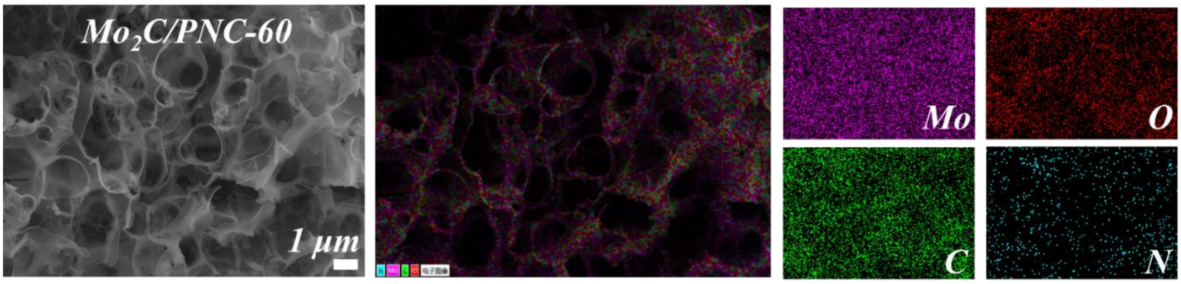

Fig. S11 SEM images and EDS element mapping (Mo, C, N, and O) of Mo_2C/PNC-60

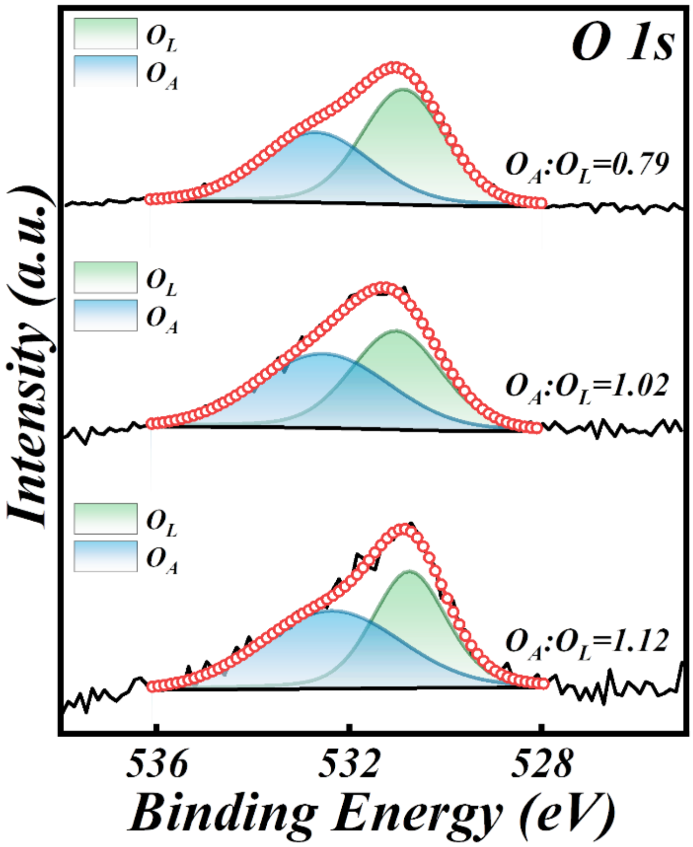

Fig. S12 XPS spectra of O 1s of $MoSe_2/MoO_2$/PNC-60, $MoSe_2$/PNC-60, and $MoSe_2$/MoC/PNC-60 (top-down)

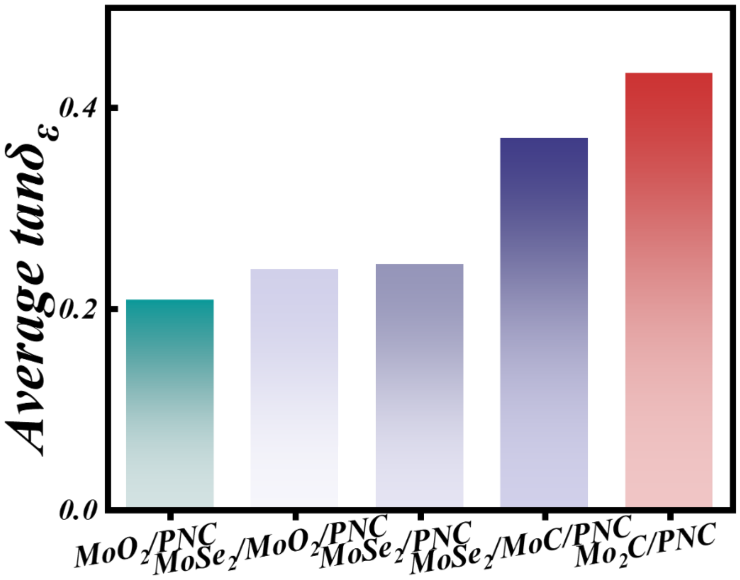

Fig. S13 Average tangent of the permittivity constant

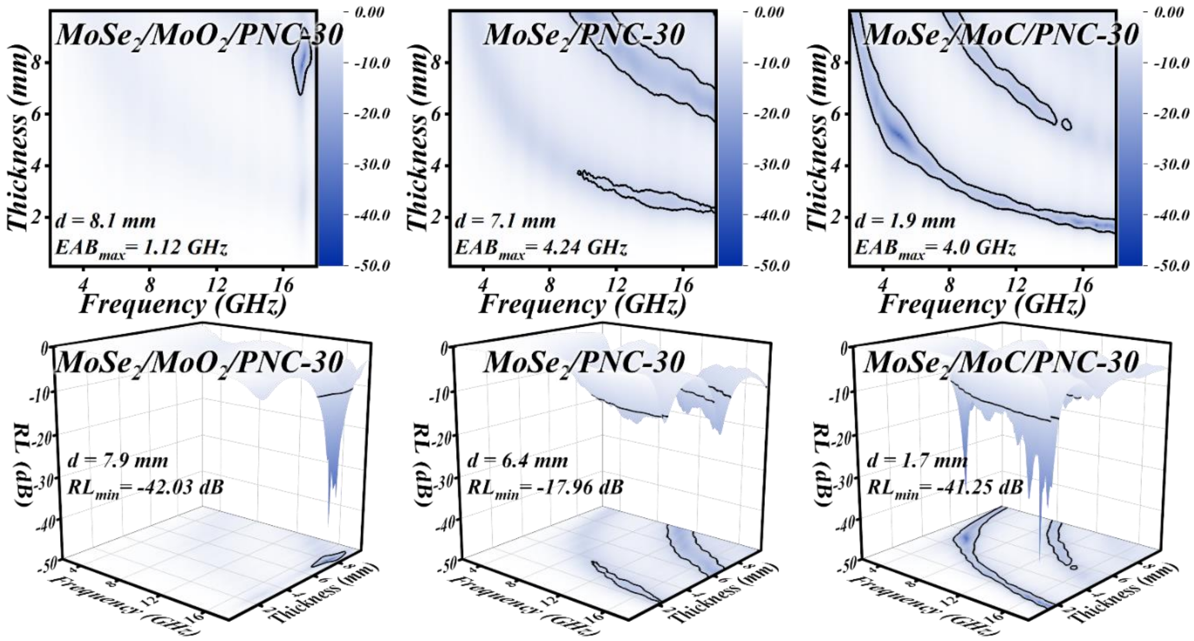

Fig. S14 2D RL and 3D RL images of each sample (x=30)

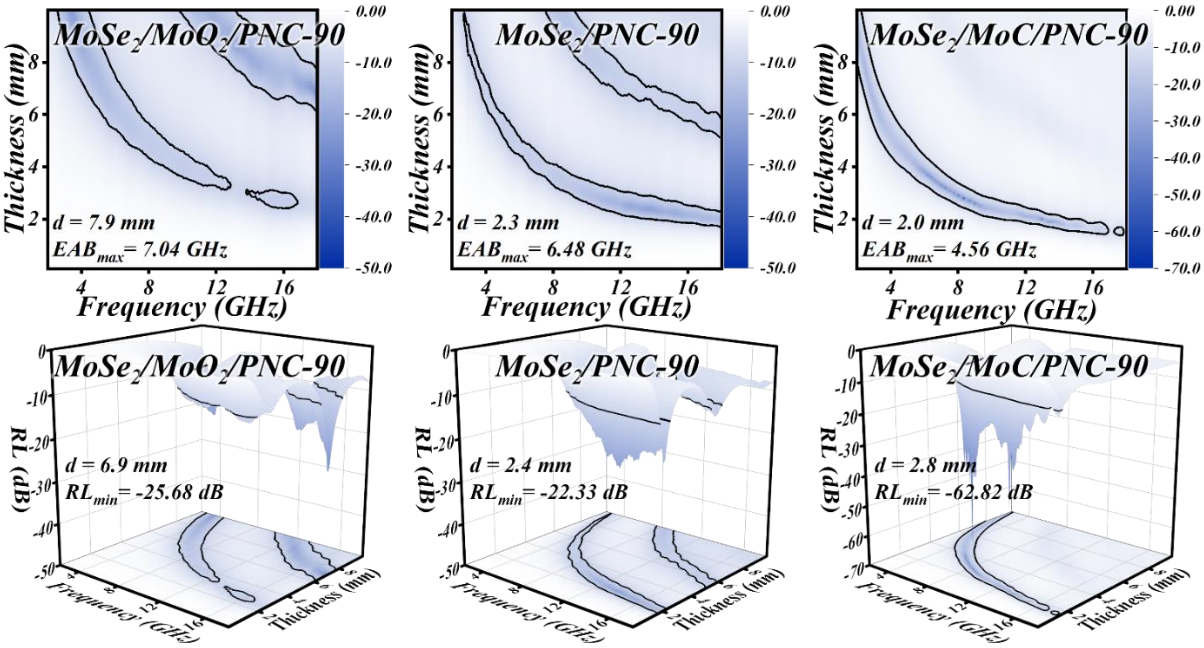

Fig. S15 2D RL and 3D RL images of each sample (x=90)

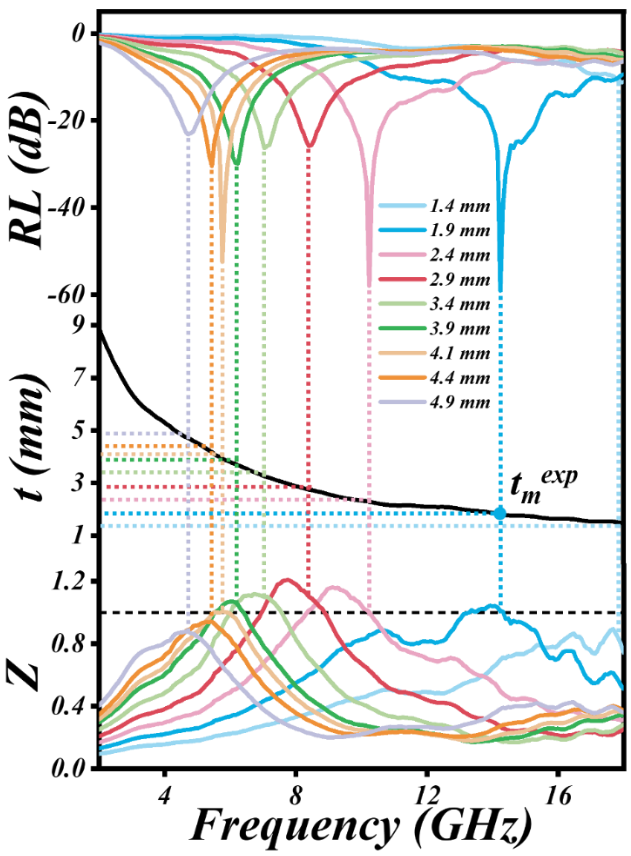

Fig. S16 The RL value changes with frequency at different matching thickness, the relationship between the simulation of the matching thickness value (t_m) and frequency (f_m) under l/4 conditions; the Z values versus frequency at different matching thickness for $MoSe_2$/MoC/PNC-60

Table S1 Mass ratio of each element obtained from EDS

	Mo wt%	Se wt%	C wt%	N wt%	O wt%	Mo/Se at%
MoSe₂/MoO₂/PNC-60	23.97	37.99	32.57	1.10	4.38	***51.92***
MoSe₂/PNC-60	22.69	37.17	32.29	1.85	6.00	***50.23***
MoSe₂/MoC/PNC-60	24.02	37.30	32.88	1.31	4.48	***52.99***

Table S2 The corrosion potential and corrosion current density of all samples

**Sample**	**E_{corr} (V vs. Ag/AgCl)**	**I_{corr} (A/cm^{-2})**
**Q235 bare steel**	-0.504	8.15×10^{-5}
**Pure epoxy**	-0.330	1.12×10^{-6}
**$MoSe_2$/MoO_2/PNC-60/epoxy**	-0.212	8.08×10^{-7}
**$MoSe_2$//PNC-60/epoxy**	-0.131	8.10×10^{-7}
**$MoSe_2$/MoC/PNC-60/epoxy**	-0.234	1.58×10^{-6}
